# HTT loss-of-function contributes to RNA deregulation in developing Huntington’s disease neurons

**DOI:** 10.1186/s13578-025-01443-5

**Published:** 2025-07-09

**Authors:** Emilia Kozłowska, Agata Ciołak, Grażyna Adamek, Julia Szcześniak, Agnieszka Fiszer

**Affiliations:** 1 Department of Medical Biotechnology, Institute of Bioorganic Chemistry, Polish Academy of Sciences, Noskowskiego Str. 12/14, Poznań, 61-704 Poland; 2 Department of Neuronal Cell Biology, Institute of Bioorganic Chemistry, Polish Academy of Sciences, Noskowskiego Str. 12/14, Poznań, 61-704 Poland

**Keywords:** Huntington’s disease, Transcription factors, miRNAs, iPSC-derived neurons, Loss-of-function mechanism

## Abstract

**Background:**

Huntington’s disease (HD) is a neurodegenerative disorder caused by the expansion of CAG repeats in the *HTT* gene, which results in a long polyglutamine tract in the huntingtin protein (HTT). One of the earliest key molecular mechanisms underlying HD pathogenesis is transcriptional dysregulation, which is already present in the developing brain. In this study, we searched for networks of deregulated RNAs crucial for initial transcriptional changes in HD- and HTT-deficient neuronal cells.

**Results:**

RNA-seq (including small RNAs) was used to analyze a set of isogenic human neural stem cells. The results were validated using additional methods, rescue experiments, and in the medium spiny neuron-like cells. We observed numerous changes in gene expression and substantial dysregulation of miRNA expression in HD and *HTT*-knockout (*HTT*-KO) cell lines. The overlapping set of genes upregulated in both HD and *HTT*-KO cells was enriched in genes associated with DNA binding and the regulation of transcription. We observed substantial upregulation of the following transcription factors: *TWIST1*,* SIX1*,* TBX1*,* TBX15*,* MSX2*,* MEOX2* and *FOXD1*. Moreover, we identified miRNAs that were consistently deregulated in HD and *HTT*-KO cells, including miR-214, miR-199, and miR-9. These miRNAs may function in the network that regulates *TWIST1* and *HTT* expression *via* a regulatory feed-forward loop in HD.

**Conclusions:**

On the basis of overlapping changes in the mRNA and miRNA profiles of HD and *HTT*-KO cell lines, we propose that transcriptional deregulation in HD at early neuronal stages is largely caused by a deficiency of properly functioning HTT rather than a typical gain-of-function mechanism.

**Supplementary Information:**

The online version contains supplementary material available at 10.1186/s13578-025-01443-5.

## Graphical abstract

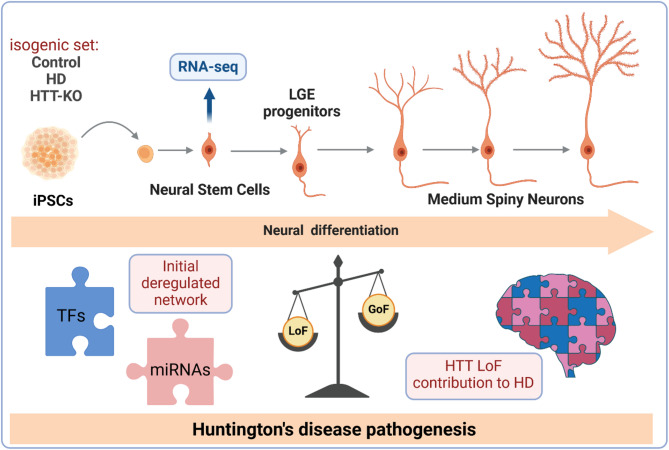

## Introduction

Huntington’s disease (HD) is an autosomal dominant neurodegenerative disorder caused by the expansion of CAG repeats in the huntingtin gene *(HTT)* [1, 2]. This mutation results in an abnormally long polyglutamine (polyQ) tract in a multifunctional protein called huntingtin (HTT). Mutant HTT (mutHTT) exhibits toxic properties, and its presence leads to the dysfunction and death of neurons, especially striatal medium spiny neurons (MSNs) [3, 4]. Despite HD manifesting at a mean age of 40 years (with cognitive and behavioral disturbances and chorea), it has begun to be recognized as a neurodevelopmental disorder [5, 6]. Early molecular alterations may contribute to the HD neurodegeneration phenotype in adults [7–10].

Although the mutHTT gain-of-function (GoF) mechanism is well recognized [11–13], the partial loss of wild-type HTT (wtHTT) may significantly contribute to the pathogenesis of HD [14] because of its role in neuronal survival [15, 16]. HTT loss-of-function (LoF) mechanisms have been widely studied in mice, and the crucial role of Htt in the embryonic stage and its important functions in adult animals have been proven [17, 18]. The complete knockout of the mouse *Htt* gene caused embryonic death [19, 20], and mice with a 50% reduction in the level of Htt presented strong malformations of the cortex and striatum [19, 21]. Additionally, mice with substantially decreased levels of wtHtt from the embryonic stage presented motor abnormalities and neurodegenerative changes [22, 23] or developed seizure disorders [24]. On the other hand, there are clear indications that the presence of mutHTT only at early stages of development leads to symptoms in adult life [25]. Therefore, for a better understanding of the initial pathogenesis of HD, the role of LoF and GoF of mutant HTT should be studied also at the early stages of neurodevelopment. Additionally, transcriptional dysregulation has been proposed to be one of the earliest and central molecular mechanisms underlying HD pathogenesis [26–28]. Experimental evidence from human tissues and HD models demonstrated massive changes in the levels of various RNAs [9, 29–31]. Some mechanisms have been suggested to explain how mutHTT or loss of wtHTT causes transcriptional dysregulation, including altered HTT interactions with positive or negative regulators of transcription [28]. In this study, we searched for initial networks of deregulated RNAs resulting from the presence of mutHTT or the absence of wtHTT in human neural cells. On the basis of a comparison of HD and *HTT*-KO cell lines, we propose that a deficiency in properly functioning HTT substantially contributes to transcriptional deregulation during neuronal differentiation in HD.

## Methods

### iPSC lines and differentiation into NSCs and neuronal cells

**Fig. 1:**
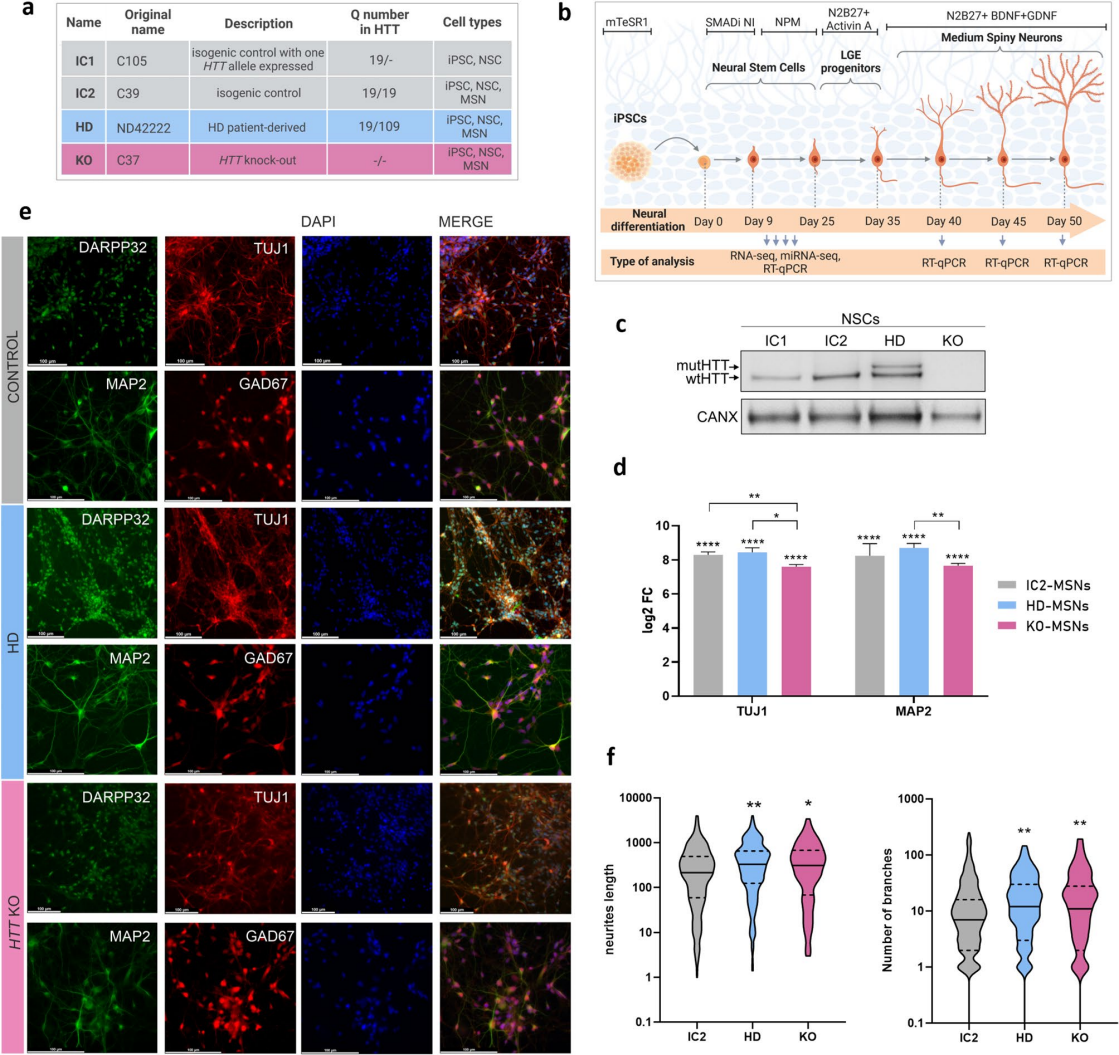
Neuronal differentiation of a set of isogenic iPSC lines **(a)** Table with information about the investigated isogenic cell lines, including the nomenclature used, the number of CAG repeats in the expressed *HTT* alleles, and the cell types used in the experiments. (**b**) Schedule of neuronal differentiation. The arrows at the bottom indicate the points at which the cells were harvested for RNA isolation. (**c**) Western blotting analysis of huntingtin levels in iPSC-derived IC1-NSCs, IC2-NSCs, HD-NSCs and KO-NSCs. Calnexin was used as a reference protein. (**d**) Relative expression of the neuronal markers *TUJ1* and *MAP2* in iPSC-derived IC2-MSNs, HD-MSNs and KO-MSNs was assessed by RT‒qPCR. FC was calculated relative to that of iPSCs via the *delta‒delta Ct* method and is shown as log2. Reference gene: *RPLP0*. Statistical analysis was performed via multiple *t tests* followed by the Holm-Sidak post hoc test. (**e**) Representative images of ICC staining for the markers MAP2, TUJ1, GAD67 and DARPP32 in the obtained neurons. Scale bar = 100 μm. (**f**) Neurite length (in arbitrary units) and number of neuronal branches quantified based on ICC staining of IC2 (*n* = 523), HD (*n* = 352), and KO (*n* = 198) MSNs. Statistical analysis was performed via one-way ANOVA followed by Dunnett’s post hoc test. *0.01 < *p* < 0,05; **p < 0.01; ***p < 0.001; ****p < 0.0001

The human HD iPSC line ND42222 was obtained from the NINDS Repository. We previously generated isogenic control lines (C105 and C39) and an *HTT*-knockout line (C37) [32]. For clarity, HD, IC1, IC2 and KO names were used in this study (Fig. 1a). IC1 was initially intended to be used as an isogenic control for all the analyses in this study but, in the meantime, we noticed that the expression of the corrected allele was inhibited in this clone, resulting in the monoallelic expression of *HTT* [33]. IC2, a control line having both *HTT* alleles transcriptionally active, was selected for further experiments. In the scope of presented study (selected TFs and miRNAs deregulation), the use of IC1 or IC2 provided consistent results for KO or HD line. Generally, HD and IC2 lines are characterized by unequal expression of two *HTT* alleles [33].

iPSCs were differentiated into NSCs using a STEMdiff SMADi Neural Induction Kit (STEMCELL Technologies) and a monolayer protocol following the manufacturer’s instructions and as we previously described [33, 34]. Differentiation of NSCs into MSN-like cells was performed according to a published protocol [35] with slight modifications, as we previously described [33]. For more details, see the Supplementary Methods.

### ICC

Neuronal cells were cultured on PDL/laminin-coated glass coverslips. First, the cells were fixed by adding 2% PFA directly to the cell culture medium and culturing the cells for 5 min, and then, the cells were gently washed with PBS and fixed with 4% PFA in PBS for 10 min. The cells were permeabilized with 0.5% Tween, blocked with 1% BSA, and then incubated with primary antibodies and fluorescent dye-conjugated secondary antibodies (all listed in Table S1). DAPI was used for nuclear staining. Images were captured with a Leica DMI6000 microscope.

Quantitation of neurites length and neuronal branches was performed using Cellprofiler (version 4.2.8). Neurons were immunostained for MAP2 to visualize neurites and counterstained with DAPI for nuclear identification. A custom analysis pipeline was developed to automatically detect nuclei and neurites, based on intensity thresholding and morphological features. Neurites were enhanced, segmented, and skeletonized to enable quantification of their total length. Nuclei served as reference points for associating neurite measurements with individual cells. Quantitative data were exported for further statistical analysis.

### Protein isolation and western blotting

For protein isolation, cells were detached with Accutase and centrifuged at 300 rpm for 4 min. The pellets were washed with PBS, lysed in PB buffer (60 mM Tris-base, 2% SDS, 10% sucrose, 2 mM PMSF) and incubated at 95 °C for 5 min. The protein concentrations were determined by measuring the absorbance at 280 nm using a DeNovix spectrophotometer. Next, 30 µg of total protein was diluted in 4x SDS loading buffer, denatured at 95 °C for 5 min and run on 3–8% NuPAGE Tris-acetate gels in NuPAGE Tris-acetate SDS Running Buffer (20x) (Thermo Fisher Scientific) at 4 °C. The proteins were wet transferred overnight onto a 0.45-µm nitrocellulose membrane (GE Healthcare) in ice-cold Towbin buffer with 0,05% SDS at 4 °C. Then, specific primary and secondary antibodies, which are listed in Table S1, were added and incubated with the membranes. Immunodetection was performed using Westar Supernova XLS3 (Cyanagen). The chemiluminescent signals on the membranes were scanned using a G: BOX documentation system (Syngene). Raw image of western blot membrane is presented in Fig. S1.

### RNA isolation

Total RNA was isolated with Direct-zol RNA Microprep (Zymo Research) according to the manufacturer’s protocol. After electroporation and transfection, total RNA was isolated with an Arcturus PicoPure RNA Isolation Kit (Thermo Fisher Scientific). For RNA-seq, RNA quality was assessed using an RNA Pico 6000 kit (Agilent) and a Bioanalyzer 2100 (Agilent).

### RNA-seq

Total RNA was isolated from 3 biological replicates of control NSCs (IC1; 4th to 6th passage) and 4 biological replicates of HD and *HTT*-KO NSCs (4th to 7th passage). RNA-seq was performed by The Genomics Core Facility in The Centre of New Technologies, University of Warsaw, Poland.

The samples were sequenced with the NovaSeq 6000 system (Illumina) with a paired-end 2 × 100 cycle procedure, 50 MR/sample for total RNA and 10 MR/sample for miRNAs. Bioinformatics analysis was performed by the IDEAS4BIOLOGY company. Sequencing libraries generation and reads analysis are described in the Supplementary Methods.

PANTHER v.17 [35] was used for Gene Ontology (GO) analysis of the DEGs (|log2FC| >1.5; padj < 0.05). Significant GO terms were determined by Fisher’s exact test after FDR correction at *p* < 0.05 and were sorted by fold enrichment.

The expression correlation of selected genes and miRNAs was calculated via the Spearman method. The correlation matrix generated with the *cor* function (*stats* R package) was visualized with corrplot v. 0.92, employing hierarchical clustering ordering.

### RT–qPCR

Reverse transcription (RT) was performed using a High-Capacity cDNA Reverse Transcription Kit (Applied Biosystems) with random primers according to the manufacturer’s protocols. RT‒qPCR was performed using SsoAdvanced Universal SYBR Green Supermix (Bio-Rad) and a CFX Connect Real-Time System (Bio-Rad) according to the manufacturer’s protocols and RT‒qPCR guidelines. On the basis of the RNA-seq data, we selected stably expressed genes, *RPLP0* and *EEF2*, for data normalization. A list of primers is provided in Table S2.

RT for miRNAs was performed using the TaqMan Advanced miRNA cDNA Synthesis Kit (Applied Biosystems). RT‒qPCR was performed using TaqMan Advanced miRNA Assays (Applied Biosystems) according to the manufacturer’s instructions. The levels of miR-92a and miR-16 were used to normalize the data.

### Electroporation of NSCs with plasmids

The HTT-19Q and HTT-109Q plasmids were pBacMam2-DiEx-LIC-C-flag_huntingtin_full-length_Q19 and pBacMam2-DiEx-LIC-C-flag_huntingtin_full-length_Q109, respectively, and were gifts from Cheryl Arrowsmith (Addgene, plasmids #111741; http://n2t.net/addgene:111741; RRID: Addgene_111741 and #111730; http://n2t.net/addgene:111730; RRID: Addgene_111730) [36].

Plasmids were isolated using the GeneJet Plasmid Maxiprep Kit (Thermo Scientific). KO-NSCs were dissociated into single-cell suspensions using Accutase, counted using a TC20 Automated Cell Counter (Bio-Rad) and electroporated with the Neon Transfection System (Invitrogen). A total of 6 × 10^5^ cells were resuspended in buffer R and 50 µg of the HTT-19Q plasmid, 50 µg of the HTT-109Q plasmid or 35 µg of the GFP plasmid and electroporated in 10 µL tips under the following conditions: 1150 V, 10 ms, 3 pulses.

### Transfection of NSCs with the miR-9 mimic

NSCs were cultured on Geltrex-treated 12-well plates in NPM for 24 h until they reached 30 to 50% confluence. Lipofectamine 2000 (Thermo Fisher) was mixed with 1, 10, or 30 nM miR-9 mimic (miRVana; cat# 4464066) or 20 nM control fluorescent BlockIT siRNA (Thermo Fisher) and applied to the NSC cultures after 20 min. After 4 h, the medium was replaced with fresh medium supplemented with 10 nM Y-27,632, and after another 24 h, the medium was replaced with medium without Y-27,632.

### Statistical analysis

The experiments were repeated at least three times. The graphs presenting the values and error bars (means ± SEMs) were generated using GraphPad Prism 8 software. p values < 0.05 were considered significant.

The representation factor (RF) was calculated via the tool provided by http://nemates.org/MA/progs/overlap_stats.html. An RF > 1 indicates more overlap than random.

### Data sharing

Raw and processed RNA-seq datasets were deposited in the Gene Expression Omnibus (GEO), with accession numbers GSE270472 (for total RNA-seq) and GSE270473 (small RNA-seq).

## Results

### Neuronal differentiation of a set of isogenic cell lines with different *HTT* variants

We aimed to reveal and compare networks of deregulated RNAs during the neural differentiation of a set of isogenic human cell lines in the context of HTT function and dysfunction. To investigate both mechanisms underlying HD, mutHTT GoF and wtHTT LoF, we used a cell line endogenously expressing *mutHTT* together with the *wtHTT* allele (named HD), control lines expressing normal *HTT* (named IC1 and IC2, which express one and two *wtHTT* alleles, respectively), and an *HTT-*knockout line (named KO) (Fig. 1a) [32]. To model neural cells from the region that is most affected in the HD brain, the striatum, we differentiated iPSCs into NSCs, then into striatal progenitors, and subsequently into MSN-like cells (Fig. 1b). We confirmed the absence of HTT in the KO-NSCs by WB and observed lower levels of wtHTT in IC1-NSCs as a consequence of inactivation of one *HTT* allele [33] (Fig. 1c). Generally, we observed some difficulties with neural differentiation of KO line (some experiments were unsuccessful). In obtained MSNs, the expression of neuronal markers *TUJ1* and *MAP2* was more than 150-fold higher in all lines, compared with those in iPSCs (Fig. 1d). Nevertheless, we observed some differences in these markers’ expression between lines - *TUJ1* expression was significantly lower in the KO-MSNs than in the other MSN lines, and *MAP2* expression was lower in the KO-MSNs than in the HD-MSNs (Fig. 1d). Moreover, the neuronal state of the obtained MSNs was confirmed *via* ICC staining for TUJ1 and MAP2, as well as for the striatal markers DARPP-32 and GAD67 (Fig. 1e). We quantified main feaures of neurites in three analyzed lines. HD- and KO-MSNs showed increased length of neurites and increased number of branches, as compared to control MSNs (Fig. 1f).

### Overlap of transcriptional changes in mut*HTT*-expressing and *HTT*-knockout neuronal cells

**Fig. 2:**
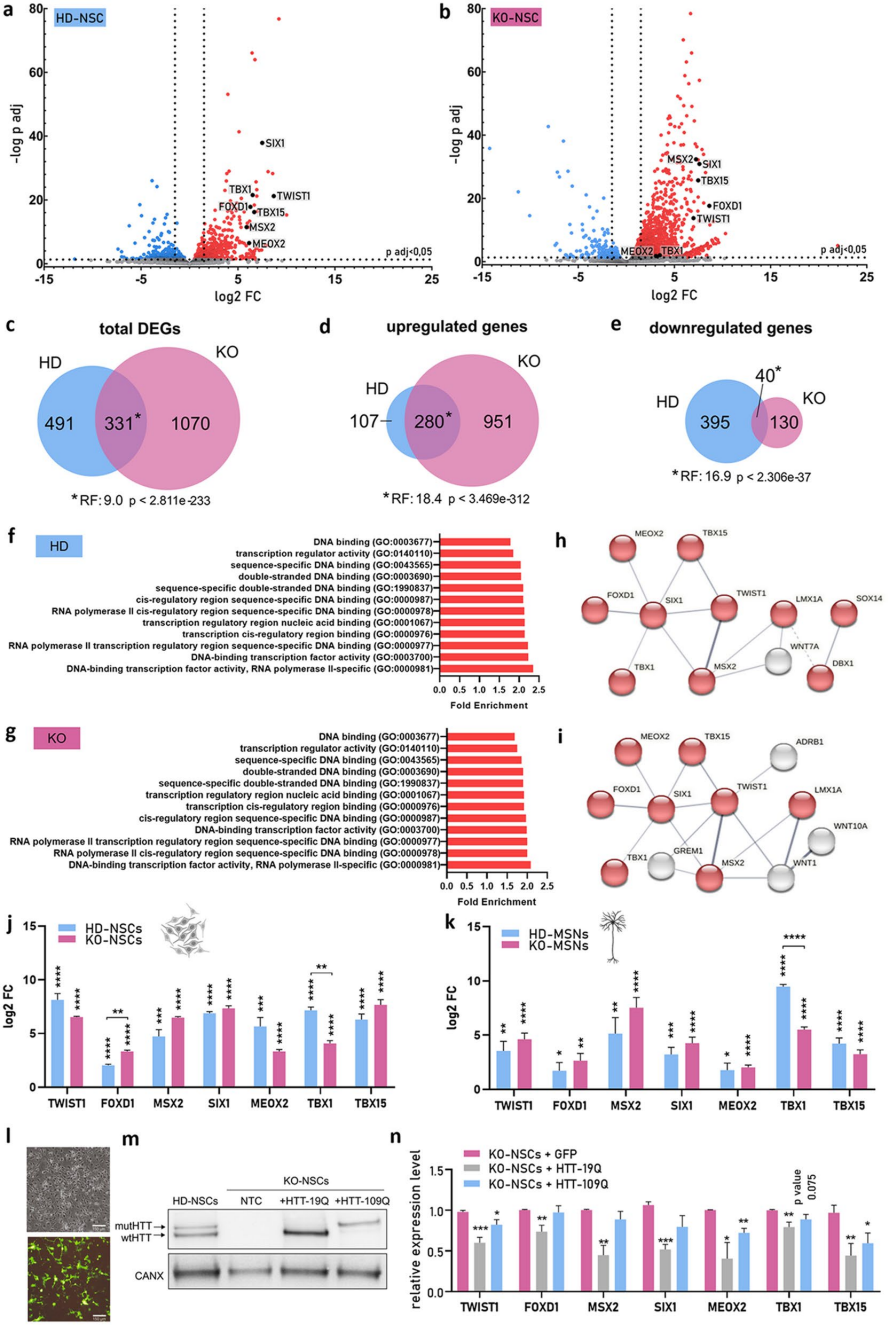
Summary of the RNA-seq results for the set of isogenic NSCs and deregulation of TFs in HD and KO neural cells (**a**, **b**) Volcano plot indicating genes with significantly increased (red dots) or decreased (blue dots) expression in HD-NSCs (**a**) and KO-NSCs (**b**) compared with IC1-NSCs. The x-axis shows log2 values of FCs in expression (vertical lines indicate a cutoff of|1.5| in this value), and the y-axis presents the–log10 of adjusted p values.TFs that were selected for validation in the later part of this study are indicated as black dots. (**c**,** d**,** e**) Venn diagrams showing the numbers of genes that were differentially expressed in HD-NSCs and KO-NSCs with total DEGs (**c**) and the separation of upregulated (**d**) and downregulated genes (**e**). (**f**, **g**) GO Panther enrichment analysis of DEGs in HD (**f**) and KO (**g**) NSCs. The selected significantly enriched GO terms are shown with false discovery rate (FDR)-corrected fold enrichment (p values < 0.05).(**h**,** i**) Prediction of the PPI network of deregulated TFs in HD (**h**) and KO (**i**) NSCs. Lines represent interactions of proteins in networks, and nodes represent proteins. Red indicates TFs related to RNA polymerase II (GO: 0000981). The FDR-corrected p values were 1.90E-04 for (**g**) and 5.22e-05 for (**i**). The figure of interaction networks was adapted from STRING v11.5. (**j**,** k**) Relative expression of TFs in HD-NSCs and KO-NSCs (vs. IC1-NSCs) (**j**) and HD-MSNs and KO-MSNs (vs. IC2-MSNs) (**k**) was analyzed via RT‒qPCR. FC was calculated relative to that of IC lines and is shown as log2. **(l**,** m**,** n)** Effect of HTT rescue on the expression of selected TFs in KO-NSCs. (**l**) Representative images of KO-NSCs 48 h after electroporation with a plasmid encoding GFP. Scale bar = 150 μm. (**m**) Western blot analysis of HTT levels in KO-NSCs 48 h after electroporation with the HTT-109Q or HTT-19Q plasmid. A sample from HD-NSCs was also loaded, and calnexin was used as a reference protein. NTC – nontreated cells. (**n**) Relative expression of TFs in KO-NSCs 48 h after electroporation with the HTT-109Q or HTT-19Q plasmid (for HTT-109Q, *n* = 5; for HTT-19Q, *n* = 4). FC was calculated relative to that of GFP-electroporated cells.Reference genes: *EEF2* and *RPLP0*. Statistical analysis was performed via multiple *t tests* (**n**) followed by the Holm-Sidak post hoc test for (**j**) and (**k**). *0.01 < *p* < 0,05; **p < 0.01; ***p < 0.001; ****p < 0.0001

RNA-seq was performed using RNA isolated from the IC1, HD, and KO NSC lines at subsequent passages. In the analyzed datasets, there was a clear separation of the HD and KO groups from the control group (Fig. S2a, b), especially the KO group presented a distinct profile from both the HD and IC1 groups (Fig. S2c). A substantially greater number of differentially expressed genes (DEGs) were identified in the KO line than in the HD line, both compared with the IC1 line (Fig. 2a-c). Specifically, we identified 1401 DEGs in KO-NSCs and 822 DEGs in HD-NSCs (|log2FC| > 1.5; padj < 0.05) (Fig. 2c; Table S3). A highly significant overlap of 331 DEGs was confirmed by a representation factor (RF) of 9 (Fig. 2c). In the HD-NSC model, a similar number of genes, 387 and 435, were upregulated and downregulated, respectively (Fig. 2d, e). In contrast, KO-NSCs were significantly different, with 1231 upregulated genes and only 170 downregulated genes (Fig. 2d, e). The HD and KO lines shared 280 upregulated and 40 downregulated genes, with highly statistically significant RFs (~ 18 and ~ 17, respectively). Notably, in the HD model, approximately 70% of the upregulated genes overlapped with those upregulated in the KO model (Fig. 2d).

GO enrichment analysis [37] revealed many disrupted pathways in HD- and KO-NSCs (Table S4). Among the DEGs in both cellular models, we observed significant enrichment of DNA binding factors, mainly TFs that are associated with RNA polymerase II (Fig. 2f, g). Analysis of the protein‒protein interaction (PPI) networks also revealed enrichment in the TF network (Fig. 2h, i). Therefore, we selected seven TFs, *TWIST1*,* FOXD1*,* MSX2*,* SIX1*,* MEOX2*,* TBX1*, and *TBX15*, which were all highly upregulated for validation (Fig. 2h, i, a and b; Table S3). All the genes, except *TBX1*, were also reported to be upregulated in brain tissues from HD patients [38], and one of them, *TWIST1*, has already been described in the context of HD [39, 40]. RT‒qPCR confirmed the significant upregulation of all 7 TFs in both HD-NSCs and KO-NSCs (Fig. 2j). Moreover, all the studied TFs were strongly upregulated in HD and KO MSN-like cells (compared with those in IC2-MSNs) (Fig. 2k).

### Rescue of *HTT* expression resulted in TFs downregulation in KO-NSCs

To confirm the impact of wtHTT or mutHTT on the expression level of the selected TFs, we rescued *HTT* expression via electroporation of KO-NSCs with plasmids expressing the full-length cDNA of human *HTT* with 19 (wtHTT) or 109 (mutHTT) CAG repeats [36]. After the optimization of plasmid delivery (Fig. 2l), we confirmed the expression of *HTT* (Fig. 2m). The delivery of a plasmid encoding normal HTT (HTT-19Q) into KO-NSCs resulted in significant downregulation of all the investigated TFs, ranging from ~ 40–60% (Fig. 2n). Moreover, after the overexpression of HTT-109Q, we observed significant downregulation of *TWIST1*,* MEOX2* and *TBX15* (~ 20–40%). This less substantial decrease may indicate that mutHTT exerted weaker effects than wtHTT did, but the impact of lower expression of mutHTT from the plasmid cannot be excluded (Fig. 2m).

### Overlapping changes in the mirnome of HD and *HTT*-KO neural cells

**Fig. 3:**
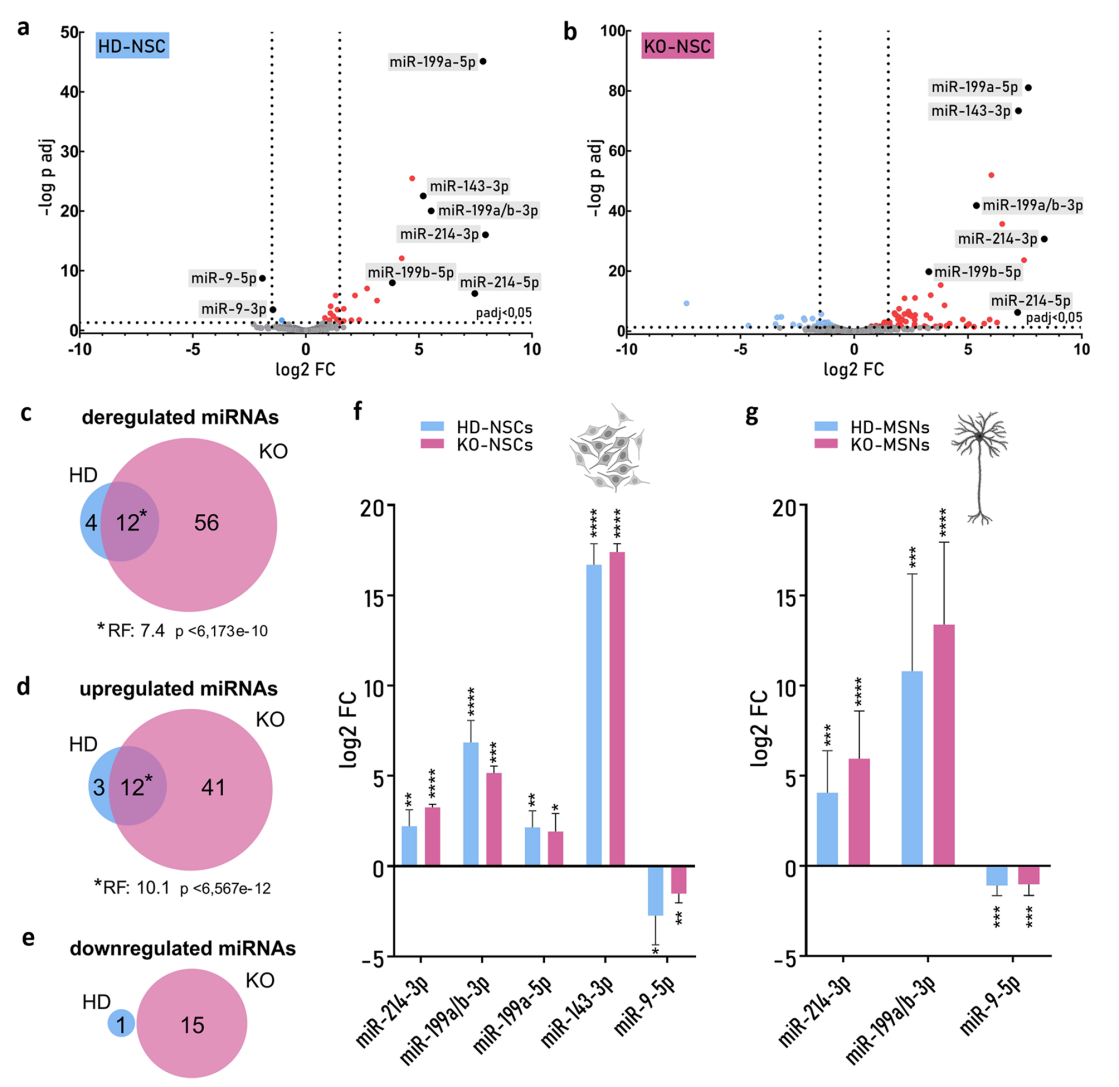
Deregulation of miRNA expression in HD and KO neural cells (**a**, **b**) Volcano plot indicating the miRNAs with significantly increased (red dots) or decreased (blue dots) expression in HD-NSCs (**a**) and KO-NSCs (**b**) versus IC1-NSCs. The x-axis shows the log2 of FCs in expression (vertical lines indicate a cutoff of |1.5| in this value), and the y-axis presents the–log10 of adjusted p values. miRNAs that were selected for validation are indicated as black dots. (**c**,** d**,** e**) Venn diagrams showing the numbers of miRNAs that were differentially expressed in HD-NSCs and KO-NSCs and the total number of differentially expressed miRNAs (**c**) and the number of upregulated (**d**) and downregulated (**e**) miRNAs. (**f**) Validation of RNA-seq data via RT‒qPCR to analyze the expression of selected miRNAs in HD-NSCs and KO-NSCs relative to that in IC1-NSCs. (**g**) Relative expression of selected miRNAs in HD-MSNs and KO-MSNs was assessed via RT‒qPCR (vs. IC2-MSNs). FC (**f**,**g**) was calculated relative to that of the IC lines via the *delta‒delta Ct* method and is shown as log2. Reference miRNAs: miR-16 and miR-92a. Statistical analysis for (**f**) and (**g**) was performed via multiple *t tests* followed by the Holm‒Sidak post hoc test. *0.01 < *p* < 0,05; **p < 0.01; ***p < 0.001; ****p < 0.0001

Numerous studies indicate that miRNA deregulation plays an important role in the pathogenesis of HD [31, 41–44]. Using small RNA sequencing, we profiled the miRNome of HD and KO-NSCs and compared it to that of IC1-NSCs (Fig. S3). We identified 16 and 68 significantly deregulated miRNAs (|log2FC|>1.5; padj < 0.05; Table S5) in HD- and KO-NSCs, respectively (Fig. 3a-c). In HD-NSCs, we identified 15 upregulated miRNAs and only 1 downregulated miRNA (Fig. 3d, e). Similar to the results of the mRNA analysis, we identified more changes in KO-NSCs than in HD-NSCs; in KO-NSCs, we identified 53 upregulated and 15 downregulated miRNAs (Fig. 3d, e). As many as 12 deregulated miRNAs were common to HD and KO cells, with a significant RF of 7.4 (Fig. 3c). We subsequently confirmed that the observed deregulation of miRNAs was unrelated to the deregulation of genes associated with the biogenesis and functioning of miRNAs (Table S6). Next, we selected for validation five of the most strongly deregulated miRNAs—miR-214-3p, miR-199a/b-3p, miR-199a-5p, and miR-143-3p—upregulated in HD-NSCs and KO-NSCs and miR-9-5p downregulated in HD-NSCs. It has already been shown that miR-143 and miR-199 are upregulated in the striatal tissues of HD patients [31] and HD mice [45], respectively. miR-214 is also known to be a posttranscriptional regulator of *HTT* expression [46, 47], and the miR-214/199a cluster is regulated by TWIST1 [48]. Additionally, miR-9 is known to be a neuronal-specific miRNA whose expression is downregulated in the brains of HD patients [49]. RT‒qPCR confirmed the strong deregulation of all the selected miRNAs. Moreover, miR-9-5p was also downregulated in KO-NSCs (Fig. 3f). We also observed strong upregulation of miR-214-3p and miR-199a/b-3p and downregulation of miR-9-5p in HD-MSNs and KO-MSNs compared with those in IC2-MSNs (Fig. 3g).

### Overexpression of miR-9-5p led to downregulation of selected TFs in HD-NSCs

**Fig. 4:**
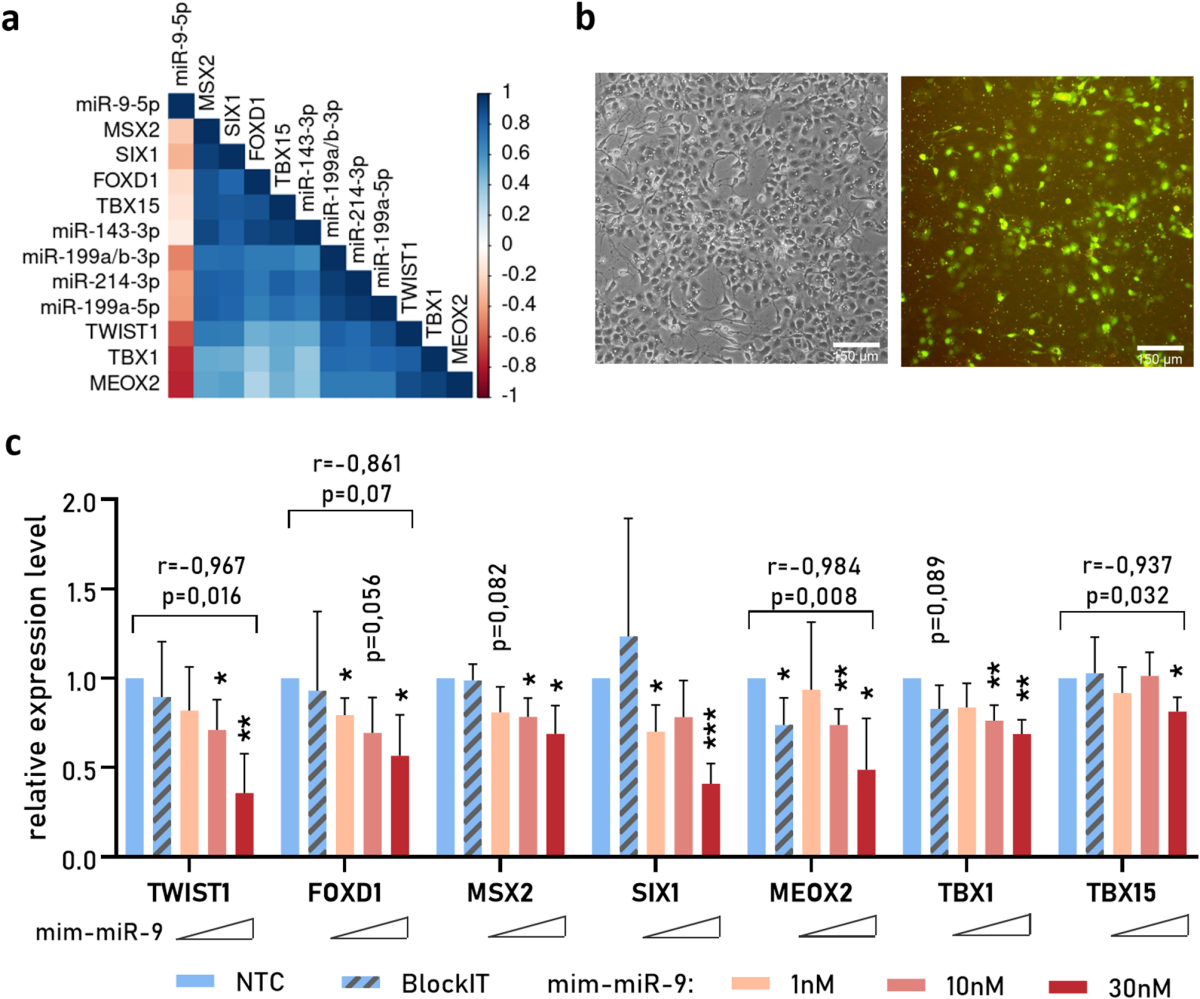
Effect of miR-9 on the expression of selected TFs in HD-NSCs (**a**) Analysis of the expression correlation of selected TFs and miRNAs. A heatmap displaying Spearman correlation coefficients calculated on the basis of TPM values from the IC1, KO and HD NSC models. (**b**) Representative images of HD-NSCs 24 h after transfection with 20 nM BlockIT control oligonucleotide. Scale bar = 150 μm. (**c**) Relative expression of selected TFs in HD-NSCs after transfection with 1 nM, 10 nM, or 30 nM miR-9 mimic. FC was calculated relative to that of nontreated cells (NTC) via the *delta‒delta Ct* method. Reference genes: *EEF2* and *RPLP0*. Statistical analysis was performed via multiple *t* tests. *0.01 < *p* < 0,05; **p < 0.01; ***p < 0.001; ****p < 0.0001. r - Pearson correlation coefficient

Analysis of RNA-seq data from NSCs revealed strong positive correlations among the expression levels of *TWIST1*, *TBX1*, and *MEOX2*, and these TFs were also negatively correlated with miR-9 levels (Fig. 4a, Fig. S4). Additionally, *MSX2*, *SIX1*, *FOXD1*, and *TBX15* formed a positively correlated network, and their expression was significantly linked to that of miR-143-3p. As expected, the expression levels of miRNAs from cluster miR199a/214 were also positively correlated.

As brain-enriched miR-9 is known to directly regulate the expression of TFs, including *TWIST1* [50], we validated this regulation by delivering a miR-9 mimic (mim-miR-9) to HD-NSCs. Transfection efficiency was monitored via the use of a FITC-labeled oligonucleotide (Fig. 4b). We observed significant dose-dependent downregulation of all 7 investigated TFs after mim-miR-9 delivery compared with nontreated cells (NTCs) (Fig. 4c). Transfection with 30 nM mim-miR-9 resulted in a decrease in the expression level of TFs to 35–80%. The downregulation of *TWIST1*,* MEOX2* and *TBX15* expression and the concentration of mim-miR-9 tended to be negatively correlated (Fig. 4c).

### Trend for mRNA and MiRNA levels to increase over time in HD neuronal cells

There are indications of increased dysregulation of *TWIST1* and miR-9 levels as HD progresses [40, 49]. In this context, we searched for progressive changes in gene expression globally in our RNA-seq data (Fig. S5, S6 and S7, Tables S7, S8, S9 and S10) and analyzed the levels of selected TFs and miRNAs during the differentiation of MSNs (Fig. S8). In HD-NSCs, we observed a high number of genes whose expression increased with increasing time in cell culture, in contrast to the HTT-KO line, which was characterized by a high number of genes whose expression decreased (see Supplementary Text for more details). These findings indicate that a progressive LoF and/or GoF of HTT in HD could be responsible for a gradual increase in TF expression. In the HTT-KO model, a lack of huntingtin may result in initial high upregulation of these TFs, which does not progress over time.

## Discussion

*HTT* mutation leads to many changes in molecular processes that, although initially subtle, have already been observed in HD patient-derived iPSCs or embryonic stem cells (ESCs) [51–54]. During the later steps of differentiation, these changes lead to disruptions that have significant implications for the functions of neural cells [9, 51, 55–61]. Stem cell-derived neural lines are models that are widely used in HD research to investigate aspects of transcriptional dysregulation [58, 62, 63], but the underlying mechanisms appear to be complex due to the interplay of the factors involved [27].

In this study, we used a unique set of human neural cell lines in the context of HTT function and dysfunction. A straightforward comparison of three cell lines (control, *HTT*-KO and HD) provided insights into the effects of HTT LoF and GoF. In the context of HTT LoF, we investigated in more detail selected deregulated RNAs that were common to both lines, HD and *HTT*-KO. We studied particular TFs and miRNAs, both of which play key roles in the regulation of gene expression at the transcriptional and posttranscriptional levels, respectively. Moreover, TFs and miRNAs expression is directly interlinked, as TFs regulate pri-miRNAs transcription, and mature miRNAs modulate the expression levels of TFs [64, 65]. The network of these interactions may easily become deregulated in the presence of an abnormal molecule, such as mutHTT, or the absence of a specific important protein, such as in the case of *HTT*-KO [11, 66–68]. wtHTT is involved in transcriptional regulation, acts as a scaffold for regulatory protein complexes in the nucleus, and interacts directly with various TFs and DNA in promoter regions [68, 69], acting as a transcriptional activator [70] or repressor [69]. Generally, changes in transcription in HD may result from the improper function of mutHTT in interactions with TF or chromatin, the occurrence of novel mutHTT interactions, or a deficiency in wtHTT, which normally regulates transcription [11, 71]. These individual mechanisms may be specific to a particular interaction, a specific cell type, or a developmental stage.

In our study, we demonstrated massive transcriptional changes in the HD and *HTT*-KO NSC lines, which surprisingly substantially overlapped (Fig. 2d). Among the genes whose expression was upregulated in HD and KO cells, we found enrichment in TFs (Fig. 2f-i). The substantial increase in the expression levels of the TFs validated in our study, namely, *TWIST1*,* FOXD1*,* MSX2*,* SIX1*,* MEOX2*, and *TBX15*, is consistent with reported changes observed in the prefrontal cortex of the brain tissue of HD patients [38]. Moreover, *TWIST1*,* SIX1*,* TBX1* and *TBX15* were also upregulated in HD iPSC-derived neurons [9], and *MEOX2* was suggested to be a suppressor gene of mutHTT toxicity in HD mouse ESCs [54]. However, among the TFs validated in our study, only *TWIST1* has been described in detail in the context of HD [39, 40]. TWIST1, which is a basic helix-loop-helix TF, is a highly conserved, antiapoptotic protein that participates in neuronal development [72]. There are two different proposed mechanisms underlying *TWIST1* upregulation in HD. Pan et al. suggested that in cortical mouse neurons, increased levels of H3K4me3 in the *Twist1* promoter are directly mediated by mutHtt [39]. On the other hand, Jen et al. proposed that in HD striatal progenitor cells, *Twist1* expression is upregulated by mutHtt through a STAT3-mediated pathway [40]. The results of these studies also led to opposite conclusions, and neurotoxic [39] or neuroprotective [40] functions of TWIST1 have been suggested. These data indicate that different mechanisms of neurodegeneration may operate in different types of cells and that the role of TWIST1 in HD should be further evaluated.

**Fig. 5:**
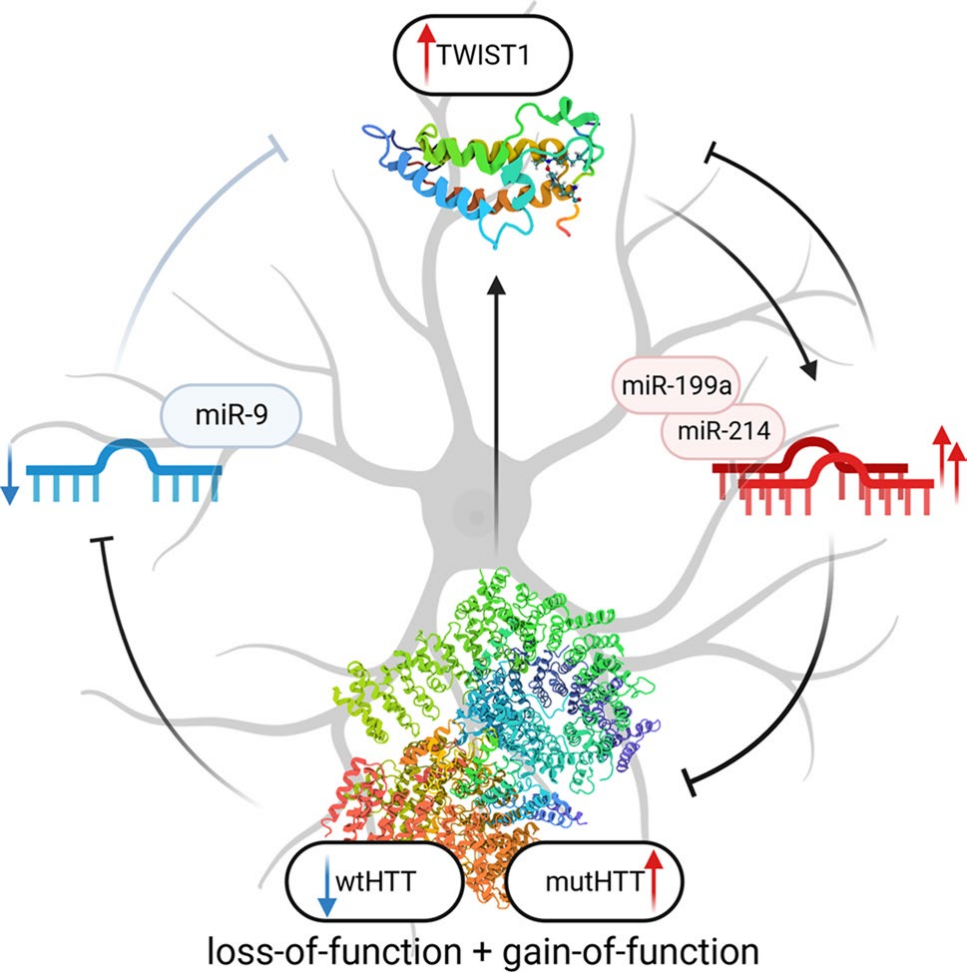
Model of the molecular network implicated in HD pathogenesis. Proposed functional network in HD neuronal cells: crosstalk mediated by a regulatory feed‒forward loop (FFL) involving HTT (due to its deficiency or dysfunction), TWIST1 TF, miR-214/199a and miR-9. For more details, see the text

Many brain-specific miRNAs are downregulated in HD [31, 49, 73]. One of these miRNAs is miR-9, which was downregulated in both our models, HD and *HTT*-KO. miR-9 is also known to directly regulate *TWIST1* expression [50], which is consistent with our RNA-seq data, which revealed a negative correlation between the expression of miR-9 and *TWIST1* in NSCs (Fig. 4a). Moreover, when we overexpressed this miRNA in HD-NSCs, we observed dose-dependent downregulation of *TWIST1* (Fig. 4c). Furthermore, TWIST1 and the miR-199a/214 cluster may regulate the expression of one another [48], and we demonstrated a significant positive correlation between these expression levels in NSCs (Fig. 4a). On the other hand, miR-214 regulates *HTT* expression [46, 47]. Therefore, we propose a network of gene regulation by TFs and miRNAs involving a regulatory feed-forward loop (FFL) that occurs in HD (Fig. 5) due to wtHTT deficiency. In healthy neurons, miR-9 contributes to the inhibition of *TWIST1* expression, and a low level of this TF leads to low expression of miR-214 and miR-199a. We also observed a significant positive correlation between the expression of miR-199a/214 and miR-143 and the TFs *SIX1*, *MSX2*, *TBX15* and *FOXD1*, which could be further investigated as previously reported.

As both mechanisms occur simultaneously in HD, investigating separate aspects of mutHTT GoF and wtHTT partial LoF is challenging. However, full HTT LoF may be easily studied in the *HTT*-KO model. In our study, the *HTT*-KO NSCs presented a more severe transcriptional phenotype than the HD cells did (Fig. 2b, d). Moreover, we observed difficulties in neural differentiation in the KO line, which was also indicated by the lower expression of *TUJ1* in the KO-MSNs than in the other lines (Fig. 1e) and supported by the lower *PAX6* expression in the KO-NSCs than in the IC1 or HD lines according to the RNA-seq data (log2FC = -2.38; KO vs. IC1, Table S3). However, compared to control neurons, we observed a subtle (but statistically significant) increase in neurite length and branching in both HD- and KO-MSNs. It may reflect early neuronal development and plasticity alterations, or compensatory mechanisms. Studies performed in stem cell-derived cortical neurons indicated decreased neurite length and branching in the case of HTT absence or downregulation [74, 75], whereas our results are consistent with those obtained for the HD line [74].

Extensive transcriptional deregulation observed in *HTT*-KO is somewhat consistent with the severe neurological phenotype observed in Lopes–Maciel–Rodan syndrome (LOMARS) patients with very low wtHTT levels [76–78]. Similarly, lower *wtHTT* expression as a result of an SNP in the *HTT* promoter region was correlated with an earlier age of HD onset [79]. Nevertheless, the effects of partial HTT LoF in HD are challenging to study, as the dominant role of mutHTT in HD has been shown, e.g., by the lack of correction of the HD phenotype by increasing levels of wtHTT protein [80, 81]. An interesting general hypothesis is that the dominant negative effect of the mutated protein on the wt protein is mediated by interference with normal functions [82, 83]. Regarding the effect of HTT LoF on HD, it can be hypothesized that the already limited function of wtHTT (due to the expression of one wt allele) becomes more limited in the presence of mutHTT. Our results support this suggestion, as more than 70% of upregulated genes in HD-NSCs were common to those upregulated in KO-NSCs (Fig. 2d). These findings indicate that increased expression of genes in HD-NSCs was mainly a direct or indirect result of a deficiency of wtHTT in the cellular regulatory network. Recently, a set of isogenic hESC lines, similar to those used in our study, was assessed in activin A-stimulation tests and neuroloid formation assays. Although different features were analyzed, the authors also reported that *HTT*-KO was characterized by an HD signature phenotype, suggesting HTT LoF component and a dominant negative mechanism of mutHTT in HD [81].

## Conclusions

The key points of this study are:

- Transcriptional and miRNome changes substantially overlap in HD and *HTT*-knockout neuronal cells,
- A network of deregulated TFs and miRNAs was identified and validated in HD and *HTT*-KO neurons,
- Loss of huntingtin function considerably contributes to the initial transcriptional deregulation in HD.

It remains to be established how initial transcriptional alterations in developing neural cells are connected with neurodevelopmental HD phenotype and whether these deregulations affect the phenotype in adult life. This aspect and investigation of the LoF of wtHTT in HD have additional important implications for the design of therapeutic strategies [16, 75, 84]. Further research should re-examine the risk of nonallele-selective therapies, and most likely, allele-selective therapies (targeting mut*HTT* expression only) will be safer for HD patients.

## Electronic supplementary material

Below is the link to the electronic supplementary material.

**Supplementary Material 1**

**Supplementary Material 2: Supplementary Fig. 1**. Raw images for western blotting. **Supplementary Fig. 2**. Clustering of NSCs samples based on RNA-seq results. **Supplementary Fig. 3**. Heatmaps of deregulated miRNAs in NSCs based on miRNA-seq results. **Supplementary Fig. 4**. Correlation of expression of selected TFs and miRNAs in IC1, HD and KO NSCs. **Supplementary Fig. 5**. Increasing and decreasing gene expression over time in NSC culture. **Supplementary Fig. 6**. GO enrichment analysis for genes classified as “increasing” in control NSCs. **Supplementary Fig. 7**. Enrichment of TFs that are associated with polymerase II in HD among „increasing genes” unique for HD-NSCs. **Supplementary Fig. 8**. Deregulation of selected TFs and miRNAs during the differentiation of HD-MSNs and KO-MSNs.

**Supplementary Material 3: Supplementary Table 1**. A list of primary and secondary antibodies with their dilutions used in immunocytochemistry and western blotting.

**Supplementary Material 4: Supplementary Table 2**. A list of primers used for RT-qPCR.

**Supplementary Material 5: Supplementary Table 3**. Lists of DEGs in HD and KO-NSCs.

**Supplementary Material 6: Supplementary Table 4**. GO enrichment analysis of DEGs in HD and KO-NSCs.

**Supplementary Material 7: Supplementary Table 5**. Lists of deregulated miRNAs in HD and KO-NSCs.

**Supplementary Material 8: Supplementary Table 6**. Analysis of miRNA biogenesis and function genes in HD-NSCs.

**Supplementary Material 9: Supplementary Table 7**. List of genes with expression increasing with time in IC1, HD and KO-NSCs.

**Supplementary Material 10: Supplementary Table 8**. List of genes with expression decreasing with time in IC1, HD and KO-NSCs.

**Supplementary Material 11: Supplementary Table 9**. GO analysis of genes increasing and decreasing in time in HD and KO-NSCs.

**Supplementary Material 12: Supplementary Table 10**. Correlation analysis of expression level of selected TFs with subsequent passages of HD and KO-NSCs.

**Supplementary Material 13:** Supplementary Text and Methods

## Data Availability

Raw and processed RNA-seq datasets were deposited in the National Center for Biotechnology Information (NCBI) Gene Expression Omnibus (GEO), with accession numbers GSE270472 (for total RNA-seq) and GSE270473 (small RNA-seq).

## Abbreviations

DEG: Differentially Expressed Genes
FC: Fold Change
FFL: Feed-Forward Loop
GO: Gene Ontology
GoF: Gain-of-Function
HD: Huntington’s Disease
HTT: Huntingtin
IC: Isogenic Control
ICC: Immunocytochemistry
iPSC: induced Pluripotent Stem Cell
KO: Knockout
LoF: Loss-of-Function
MSN: Medium Spiny Neuron
NSC: Neural Stem Cell
NTC: Nontreated Control
mutHTT: Mutant Huntingtin
polyQ: polyglutamine
RF: Representation Factor
PPI: Protein‒Protein Interaction
Q: Glutamine
TF: Transcription Factor
TPM: Transcripts Per Million
wtHTT: wild-type Huntingtin
