# Supplementary material for: HTT loss-of-function contributes to RNA deregulation in developing Huntington’s disease neurons
Source: Cell Biosci. 2025 Jul 9;15:100. doi: 10.1186/s13578-025-01443-5 (PMC12239503; doi:10.1186/s13578-025-01443-5)
Supplement: Supplementary file 1 — Supplementary Material 1 [file 13578_2025_1443_MOESM1_ESM.pdf]

a

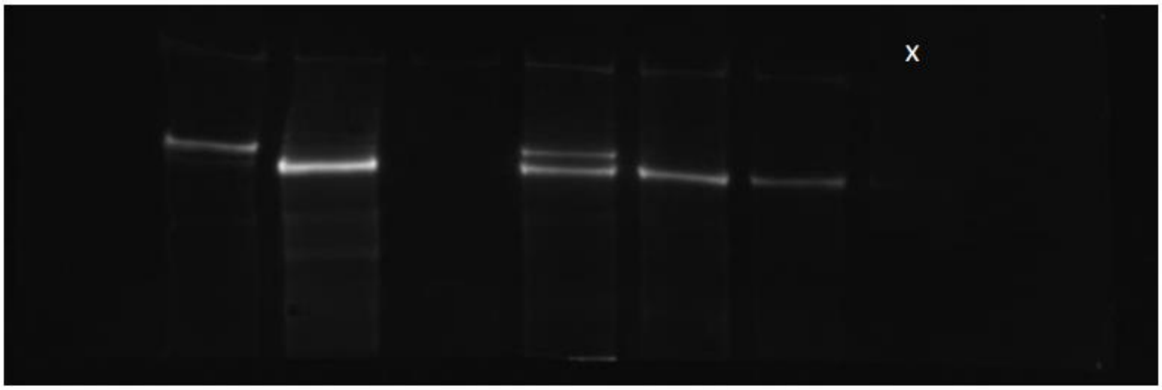

b

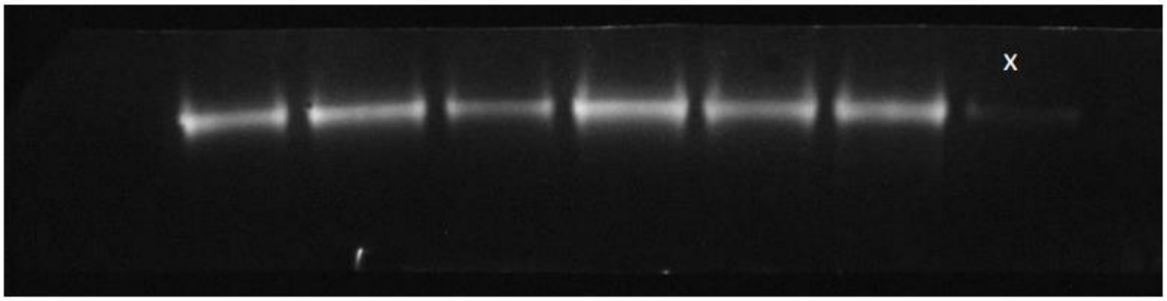

Raw images for western blotting (a) huntingtin detection, (b) calnexin detection, presented in Fig. 1c and Fig. 2m
