# Supplementary material for: HTT loss-of-function contributes to RNA deregulation in developing Huntington’s disease neurons
Source: Cell Biosci. 2025 Jul 9;15:100. doi: 10.1186/s13578-025-01443-5 (PMC12239503; doi:10.1186/s13578-025-01443-5)
Supplement: Supplementary file 2 — Supplementary Material 2: Supplementary Fig. 1. Raw images for western blotting. Supplementary Fig. 2. Clustering of NSCs samples based on RNA-seq results. Supplementary Fig. 3. Heatmaps of deregulated miRNAs in NSCs based on miRNA-seq results. Supplementary Fig. 4. Correlation of expression of selected TFs and miRNAs in IC1, HD and KO NSCs. Supplementary Fig. 5. Increasing and decreasing gene expression over time in NSC culture. Supplementary Fig. 6. GO enrichment analysis for genes classified as “increasing” in control NSCs. Supplementary Fig. 7. Enrichment of TFs that are associated with polymerase II in HD among „increasing genes” unique for HD-NSCs. Supplementary Fig. 8. Deregulation of selected TFs and miRNAs during the differentiation of HD-MSNs and KO-MSNs. [file 13578_2025_1443_MOESM2_ESM.pdf]

SUPPLEMENTARY FIGURES

a

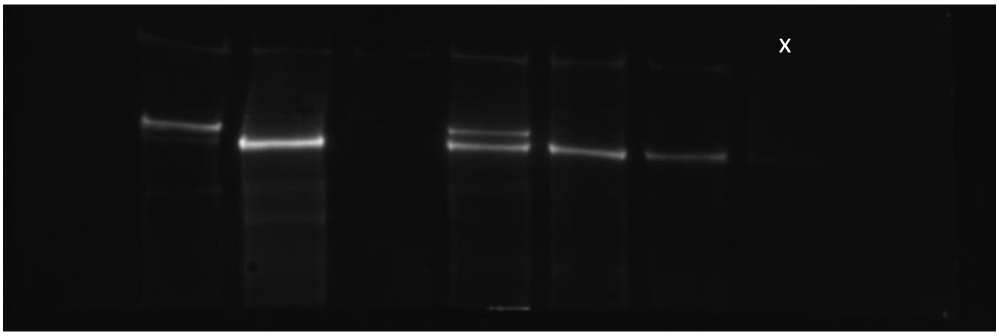

b

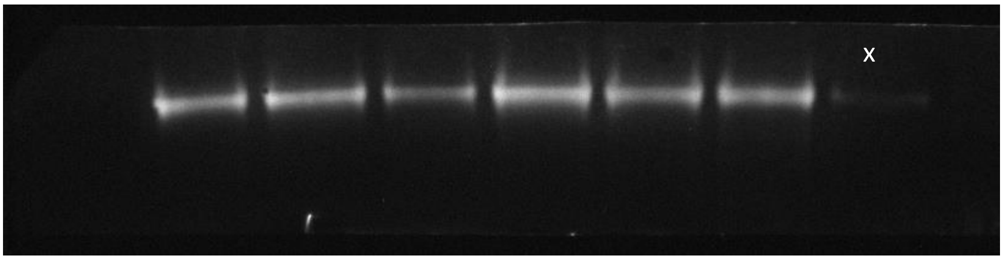

Supplementary Figure 1. Raw images for western blotting

(**a**) huntingtin detection, (**b**) calnexin detection, presented in Fig. 1c and Fig. 2m

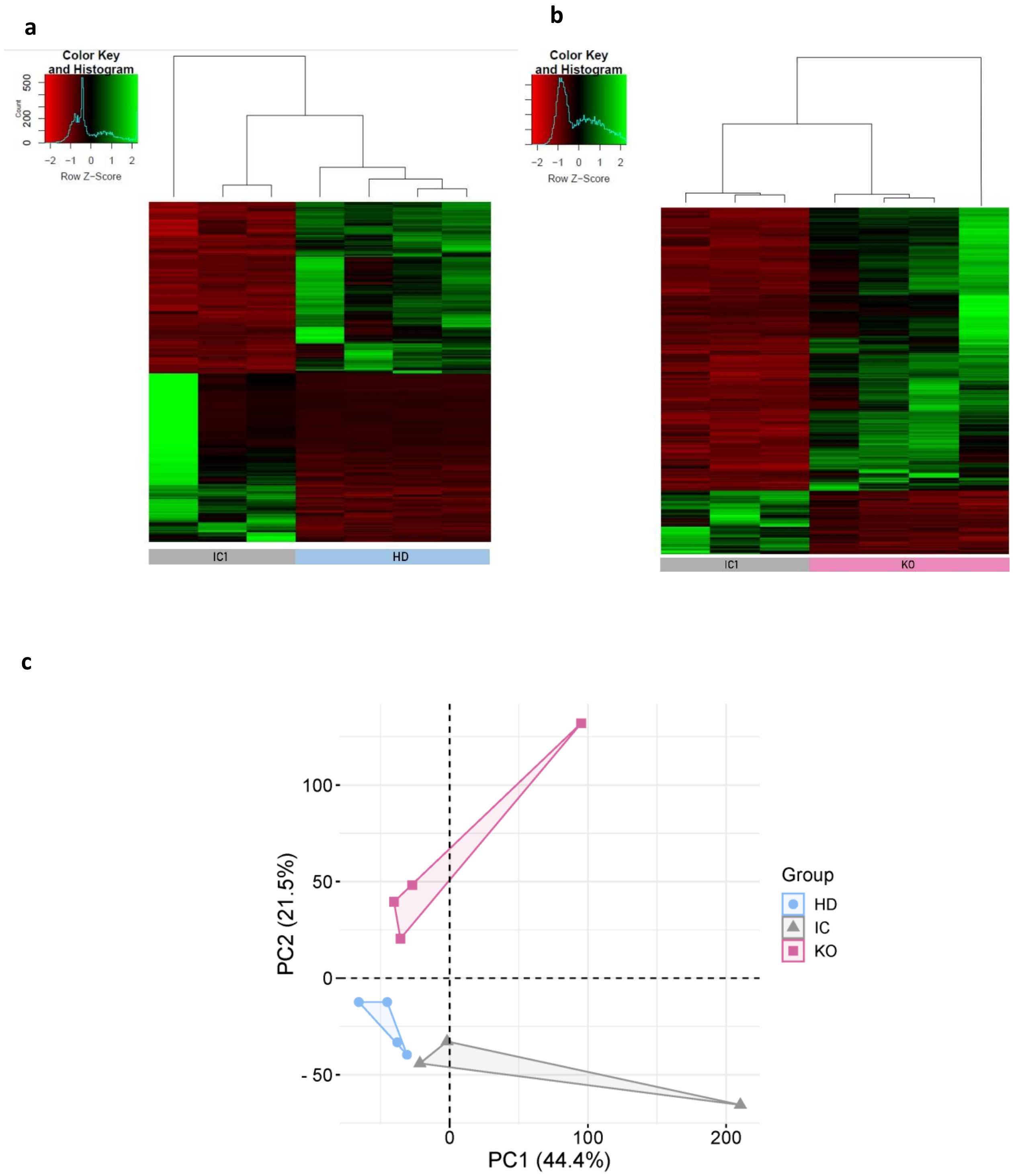

Supplementary Figure 2. Clustering of NSCs samples based on RNA-seq results

(**a**), (**b**) Heatmaps representing relative expression levels of the DEGs common to control (IC1) versus HD (a) and KO (b), with corrected padj < 0.05. Samples (in columns) and genes (in rows) are clustered by similarity. Shades of green represent upregulation, shades of red represent downregulation.

(**c**) Principal component analysis (PCA) of IC, HD and KO samples with a cutoff of 20 TPM for the sum of expression values in these samples.

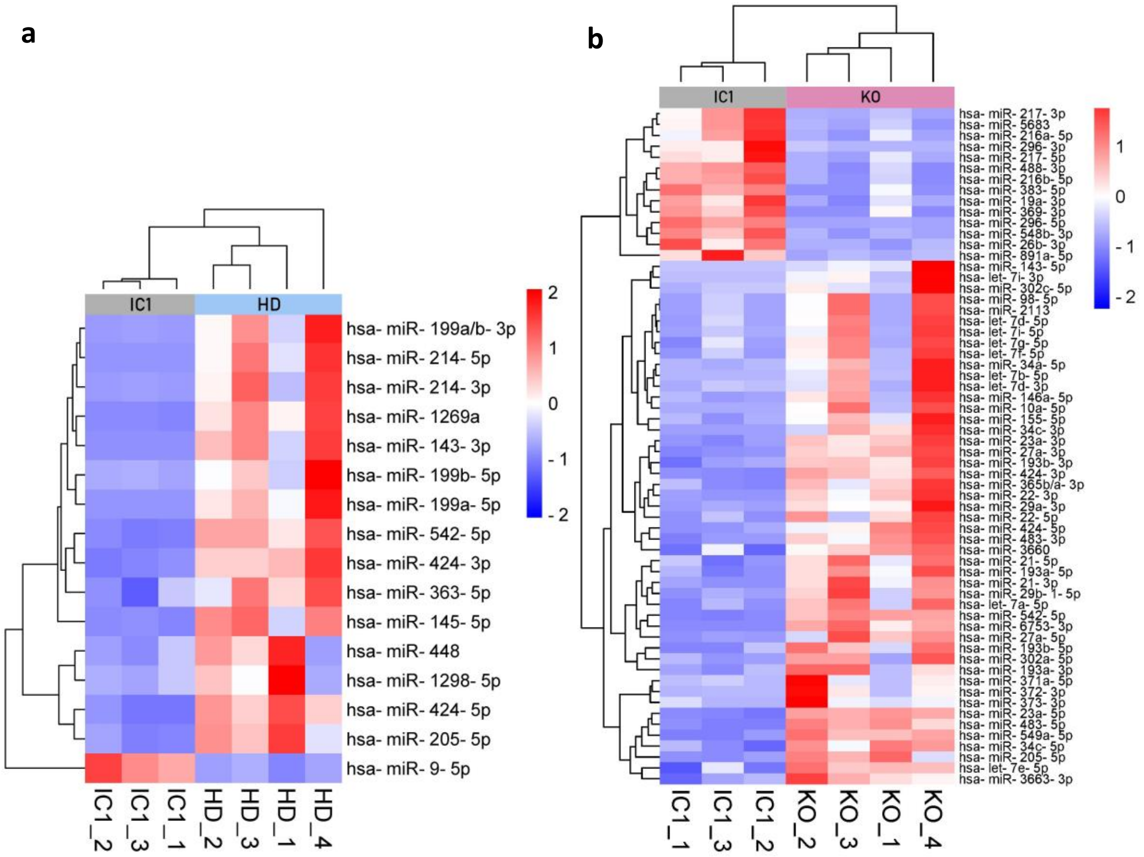

Supplementary Figure 3. Heatmaps of deregulated miRNAs in NSCs based on miRNA-seq results

(**a**), (**b**) Heatmaps representing relative expression levels of the DE miRNAs common to control (IC1) versus HD (a) and KO (b), with log2FC| >1.5 and corrected padj < 0.05. Samples (in columns) and genes (in rows) are clustered by similarity. Shades of red represent upregulation, shades of blue represent downregulation.

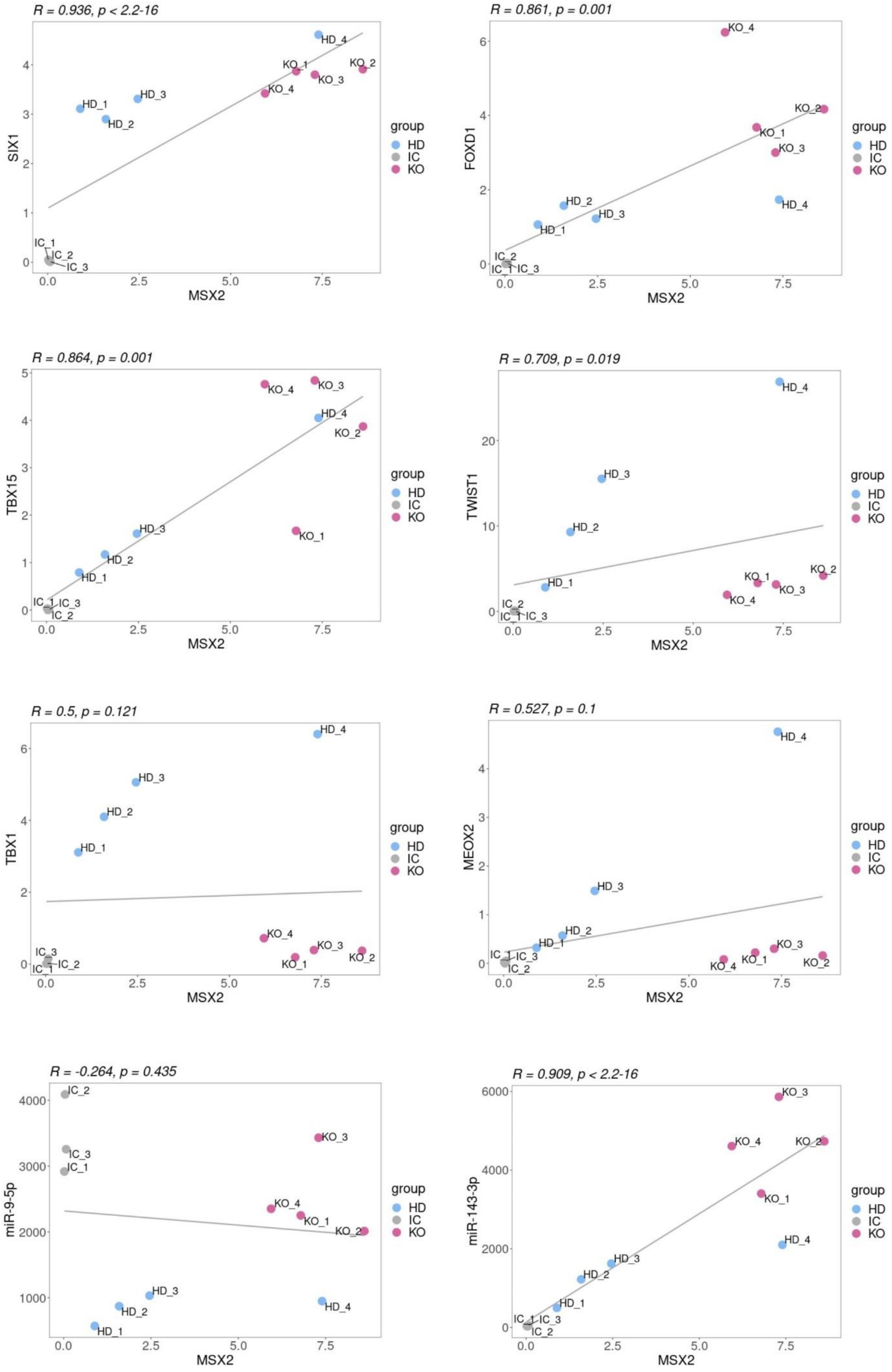

R = 0.936, p < 2.2-16
SIX1
MSX2
HD_1
HD_2
HD_3
HD_4
KO_1
KO_2
KO_3
KO_4
IC_1
IC_2
IC_3
group
HD
IC
KO
R = 0.861, p = 0.001
FOXD1
MSX2
R = 0.864, p = 0.001
TBX15
MSX2
R = 0.709, p = 0.019
TWIST1
MSX2
R = 0.5, p = 0.121
TBX1
MSX2
R = 0.527, p = 0.1
MEOX2
MSX2
R = -0.264, p = 0.435
miR-9-5p
MSX2
R = 0.909, p < 2.2-16
miR-143-3p
MSX2

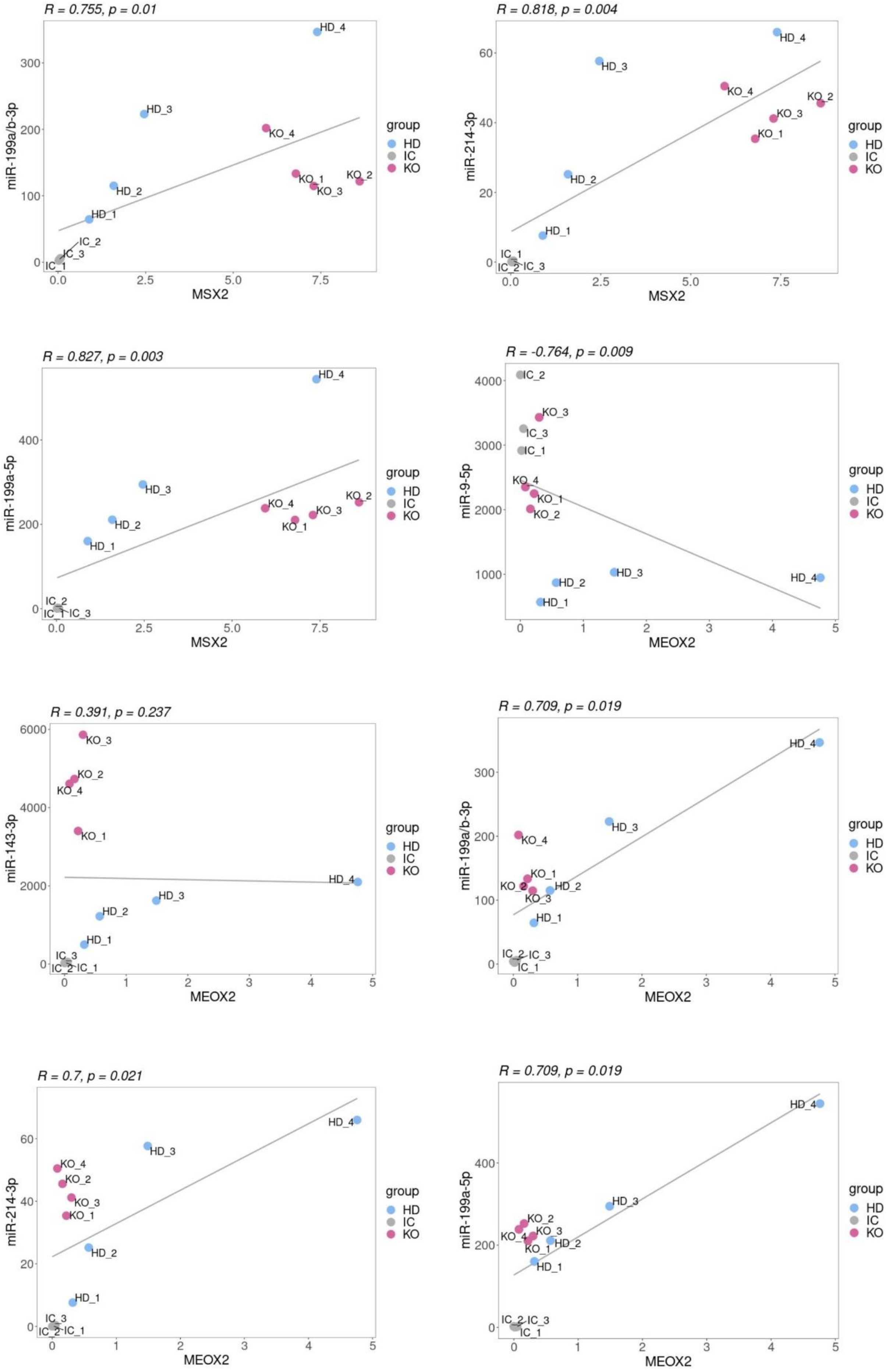
R = 0.755, p = 0.01
miR-199a/b-3p
MSX2
R = 0.818, p = 0.004
miR-214-3p
MSX2
R = 0.827, p = 0.003
miR-199a-5p
MSX2
R = -0.764, p = 0.009
miR-9-5p
MEOX2
R = 0.391, p = 0.237
miR-143-3p
MEOX2
R = 0.709, p = 0.019
miR-199a/b-3p
MEOX2
R = 0.7, p = 0.021
miR-214-3p
MEOX2
R = 0.709, p = 0.019
miR-199a-5p
MEOX2
group
HD
IC
KO
HD_1
HD_2
HD_3
HD_4
IC_1
IC_2
IC_3
KO_1
KO_2
KO_3
KO_4

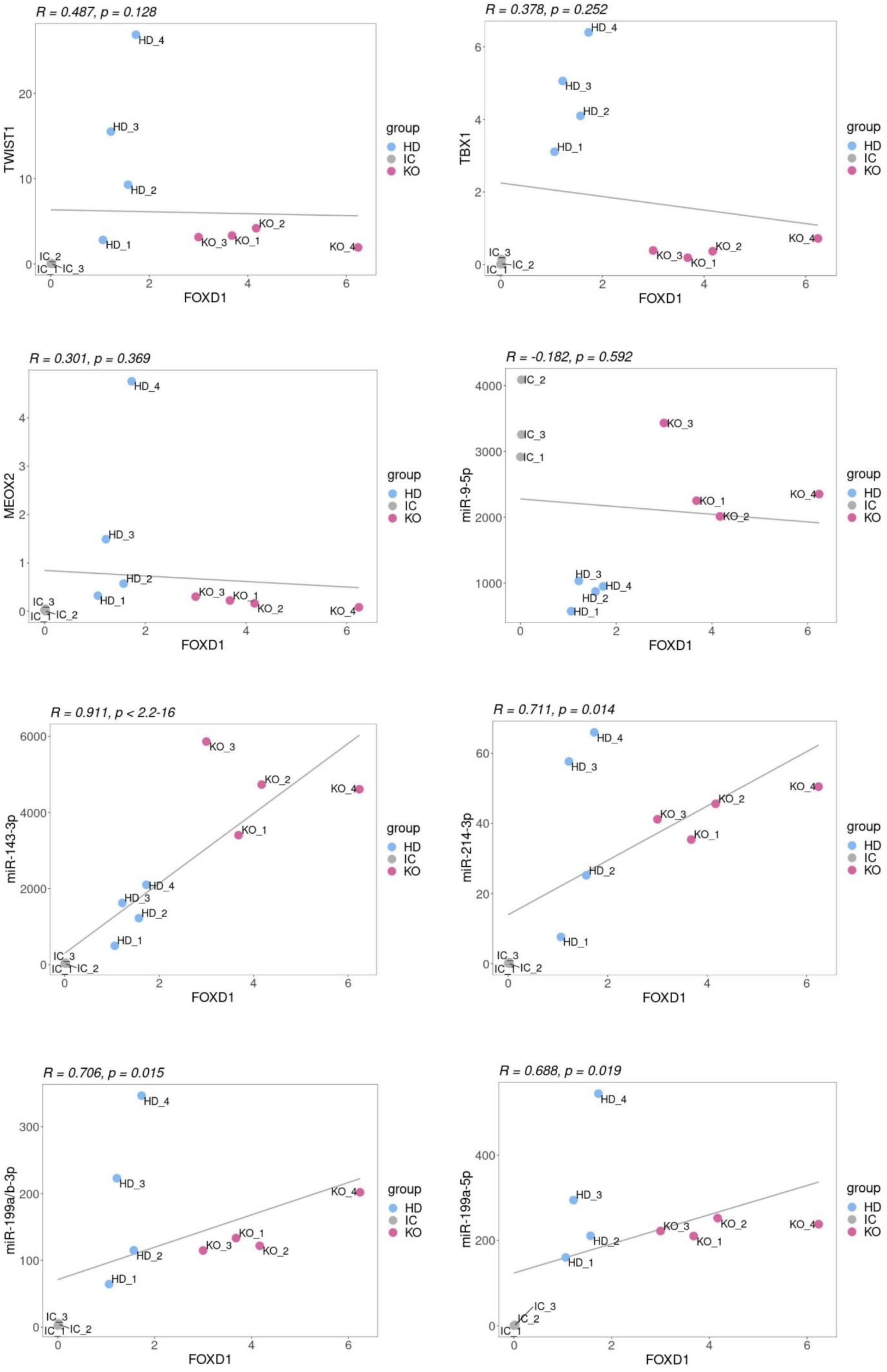
R = 0.487, p = 0.128
TWIST1
FOXD1
R = 0.378, p = 0.252
TBX1
FOXD1
R = 0.301, p = 0.369
MEOX2
FOXD1
R = -0.182, p = 0.592
miR-9-5p
FOXD1
R = 0.911, p < 2.2-16
miR-143-3p
FOXD1
R = 0.711, p = 0.014
miR-214-3p
FOXD1
R = 0.706, p = 0.015
miR-199a/b-3p
FOXD1
R = 0.688, p = 0.019
miR-199a-5p
FOXD1
group
HD
IC
KO

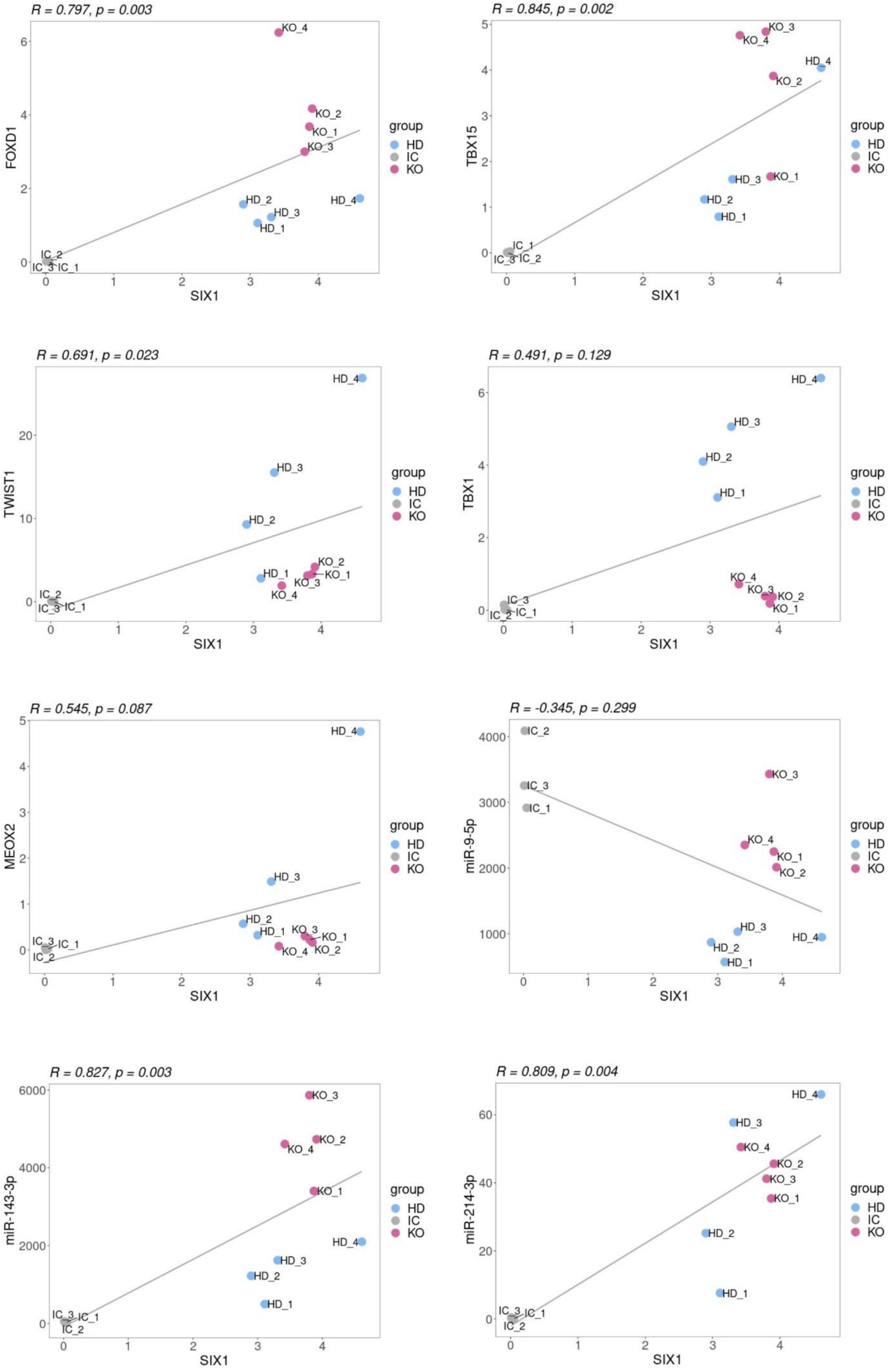
R = 0.797, p = 0.003
FOXD1
SIX1
KO_4
KO_2
KO_1
KO_3
HD_2
HD_3
HD_1
HD_4
IC_2
IC_3
IC_1
group
HD
IC
KO
R = 0.845, p = 0.002
TBX15
SIX1
R = 0.691, p = 0.023
TWIST1
SIX1
R = 0.491, p = 0.129
TBX1
SIX1
R = 0.545, p = 0.087
MEOX2
SIX1
R = -0.345, p = 0.299
miR-9-5p
SIX1
R = 0.827, p = 0.003
miR-143-3p
SIX1
R = 0.809, p = 0.004
miR-214-3p
SIX1

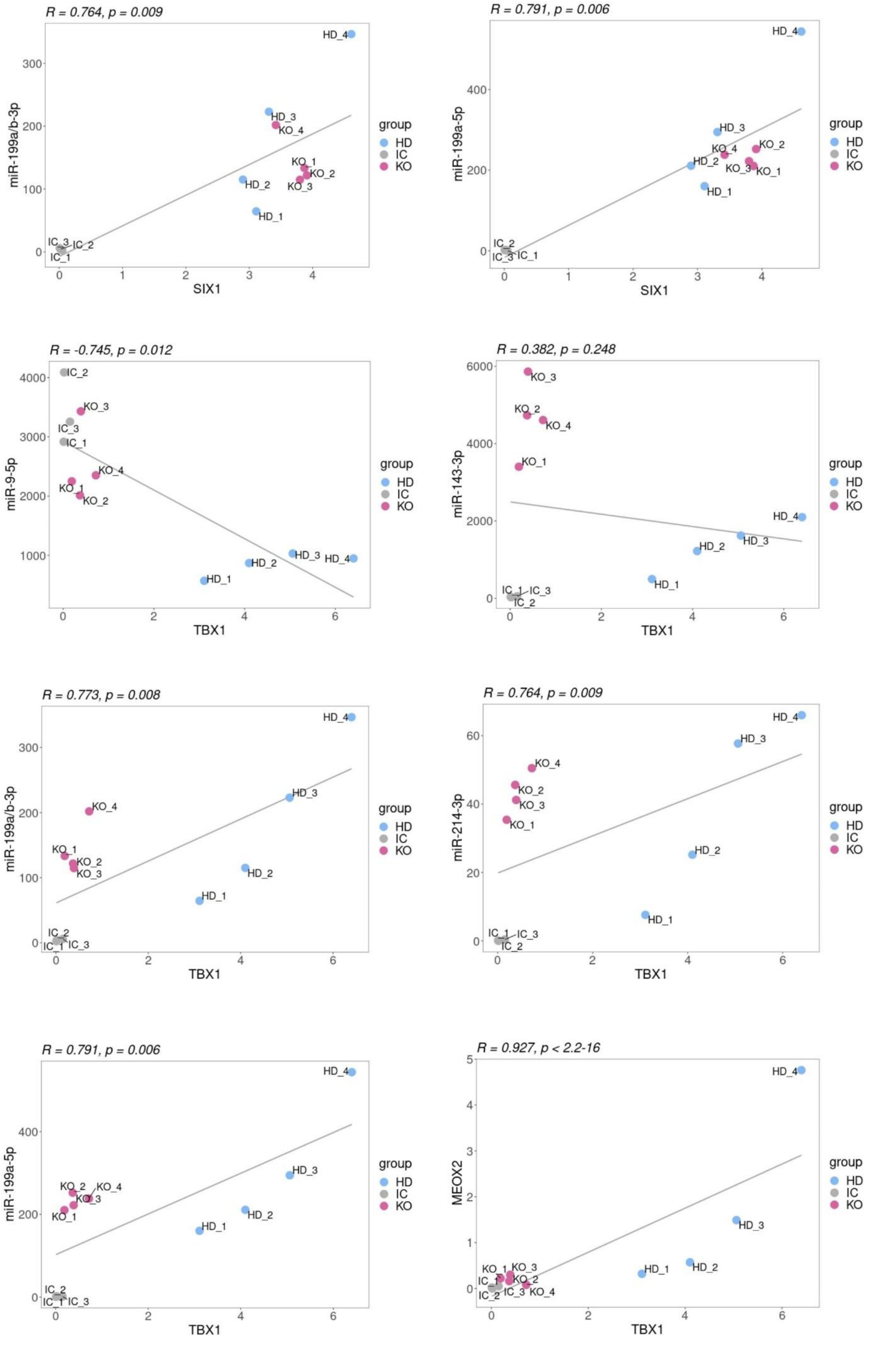
R = 0.764, p = 0.009
miR-199a/b-3p
SIX1
R = 0.791, p = 0.006
miR-199a-5p
SIX1
R = -0.745, p = 0.012
miR-9-5p
TBX1
R = 0.382, p = 0.248
miR-143-3p
TBX1
R = 0.773, p = 0.008
miR-199a/b-3p
TBX1
R = 0.764, p = 0.009
miR-214-3p
TBX1
R = 0.791, p = 0.006
miR-199a-5p
TBX1
R = 0.927, p < 2.2-16
MEOX2
TBX1
group
HD
IC
KO

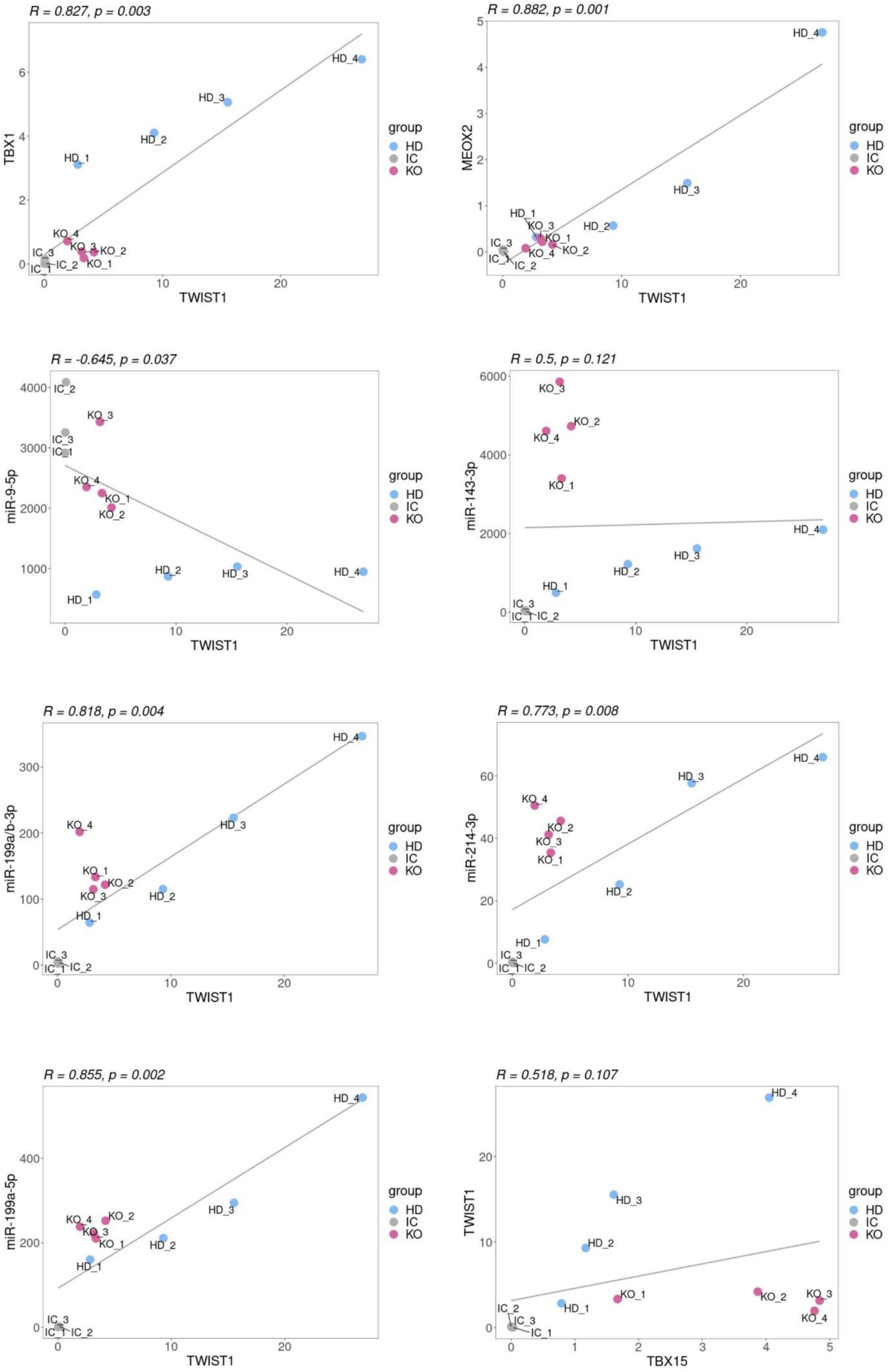
R = 0.827, p = 0.003
TBX1
TWIST1
group
HD
IC
KO
R = 0.882, p = 0.001
MEOX2
TWIST1
R = -0.645, p = 0.037
miR-9-5p
TWIST1
R = 0.5, p = 0.121
miR-143-3p
TWIST1
R = 0.818, p = 0.004
miR-199a/b-3p
TWIST1
R = 0.773, p = 0.008
miR-214-3p
TWIST1
R = 0.855, p = 0.002
miR-199a-5p
TWIST1
R = 0.518, p = 0.107
TWIST1
TBX15

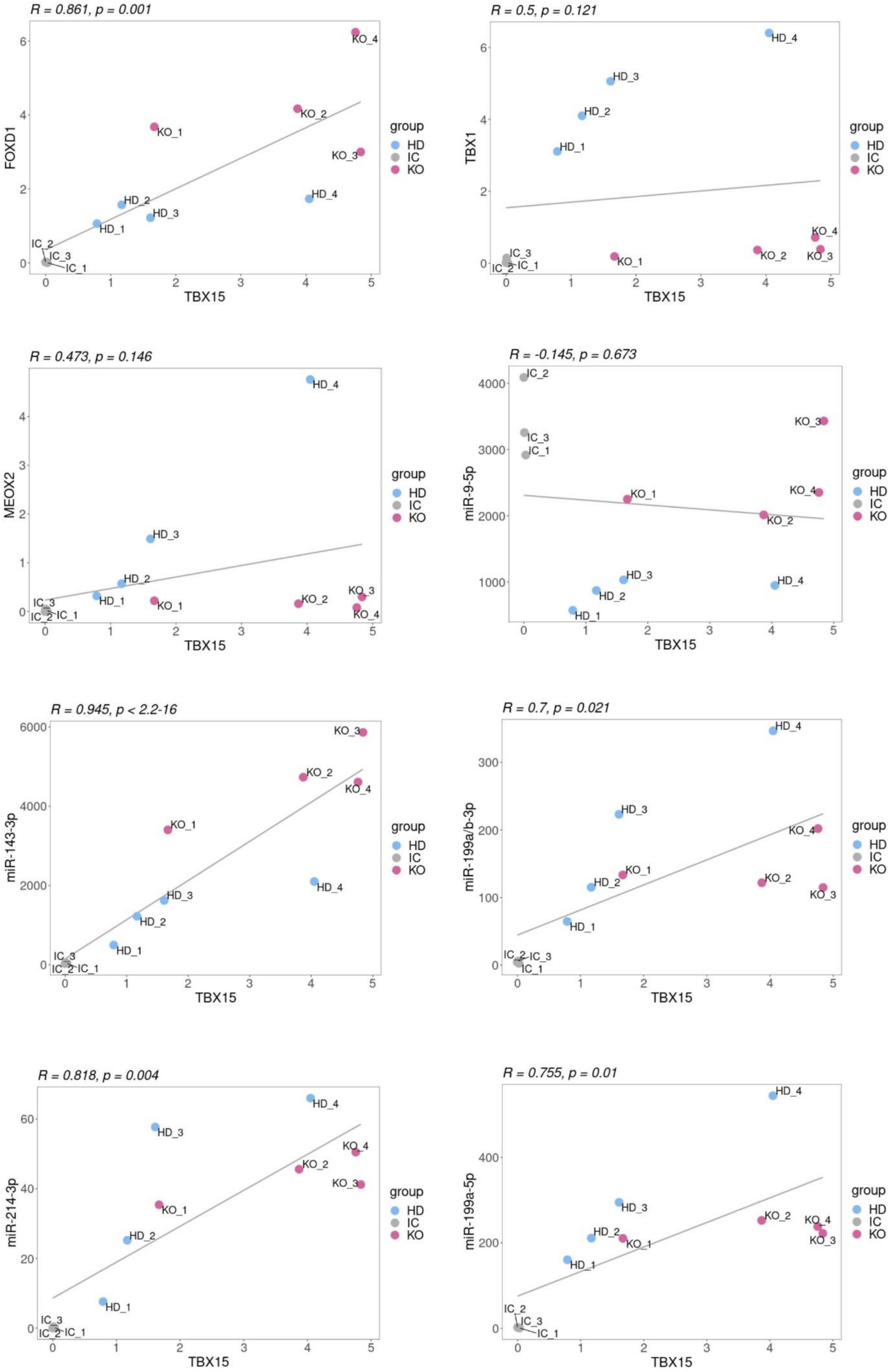
R = 0.861, p = 0.001
FOXD1
TBX15
R = 0.5, p = 0.121
TBX1
TBX15
R = 0.473, p = 0.146
MEOX2
TBX15
R = -0.145, p = 0.673
miR-9-5p
TBX15
R = 0.945, p < 2.2-16
miR-143-3p
TBX15
R = 0.7, p = 0.021
miR-199a/b-3p
TBX15
R = 0.818, p = 0.004
miR-214-3p
TBX15
R = 0.755, p = 0.01
miR-199a-5p
TBX15
group
HD
IC
KO
HD_1
HD_2
HD_3
HD_4
IC_1
IC_2
IC_3
KO_1
KO_2
KO_3
KO_4

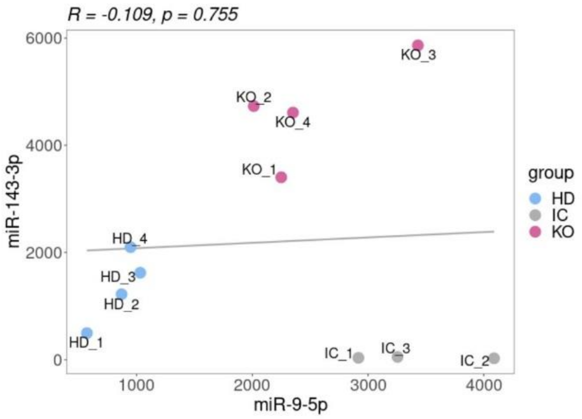
R = -0.109, p = 0.755
miR-143-3p
miR-9-5p
group
HD
IC
KO
KO_3
KO_2
KO_4
KO_1
HD_4
HD_3
HD_2
HD_1
IC_1
IC_3
IC_2

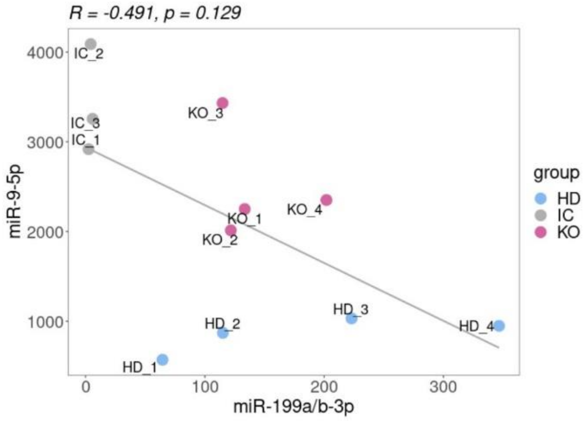
R = -0.491, p = 0.129
miR-9-5p
miR-199a/b-3p
group
HD
IC
KO
IC_2
IC_3
IC_1
KO_3
KO_4
KO_1
KO_2
HD_2
HD_3
HD_4
HD_1

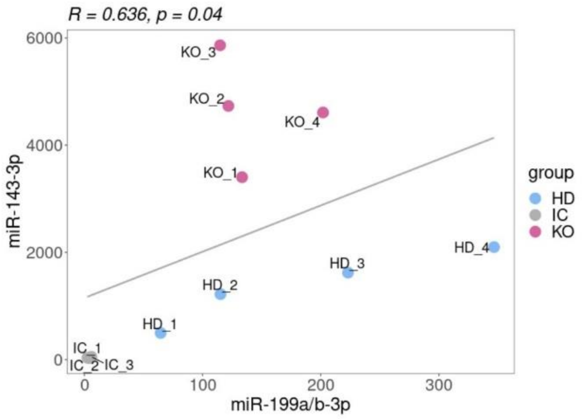
R = 0.636, p = 0.04
miR-143-3p
miR-199a/b-3p
group
HD
IC
KO
KO_3
KO_2
KO_4
KO_1
HD_4
HD_3
HD_2
HD_1
IC_1
IC_2
IC_3

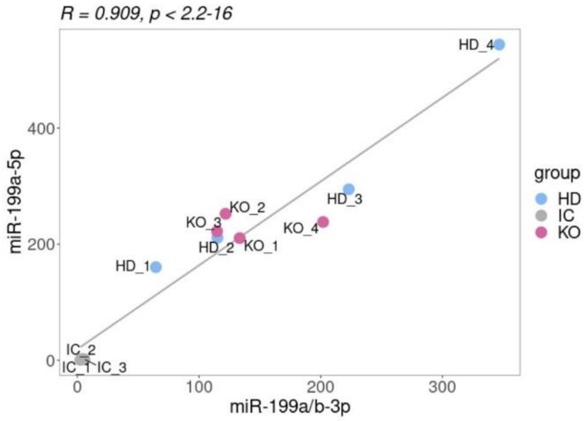
R = 0.909, p < 2.2-16
miR-199a-5p
miR-199a/b-3p
group
HD
IC
KO
HD_4
HD_3
KO_2
KO_3
KO_4
HD_2
KO_1
HD_1
IC_2
IC_1
IC_3

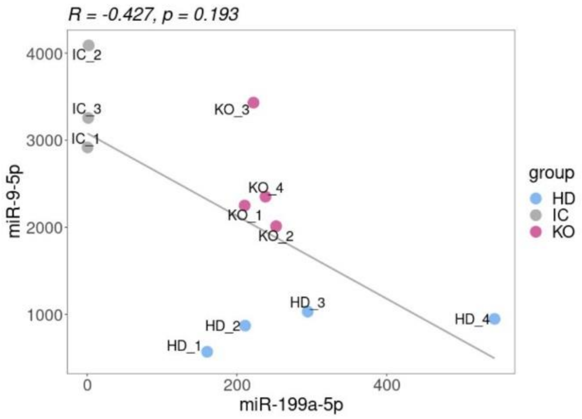
R = -0.427, p = 0.193
miR-9-5p
miR-199a-5p
group
HD
IC
KO
IC_2
IC_3
IC_1
KO_3
KO_4
KO_1
KO_2
HD_3
HD_2
HD_4
HD_1

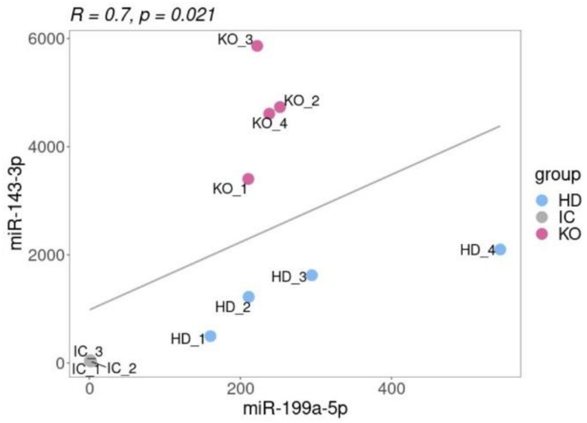
R = 0.7, p = 0.021
miR-143-3p
miR-199a-5p
group
HD
IC
KO
KO_3
KO_2
KO_4
KO_1
HD_4
HD_3
HD_2
HD_1
IC_3
IC_1
IC_2

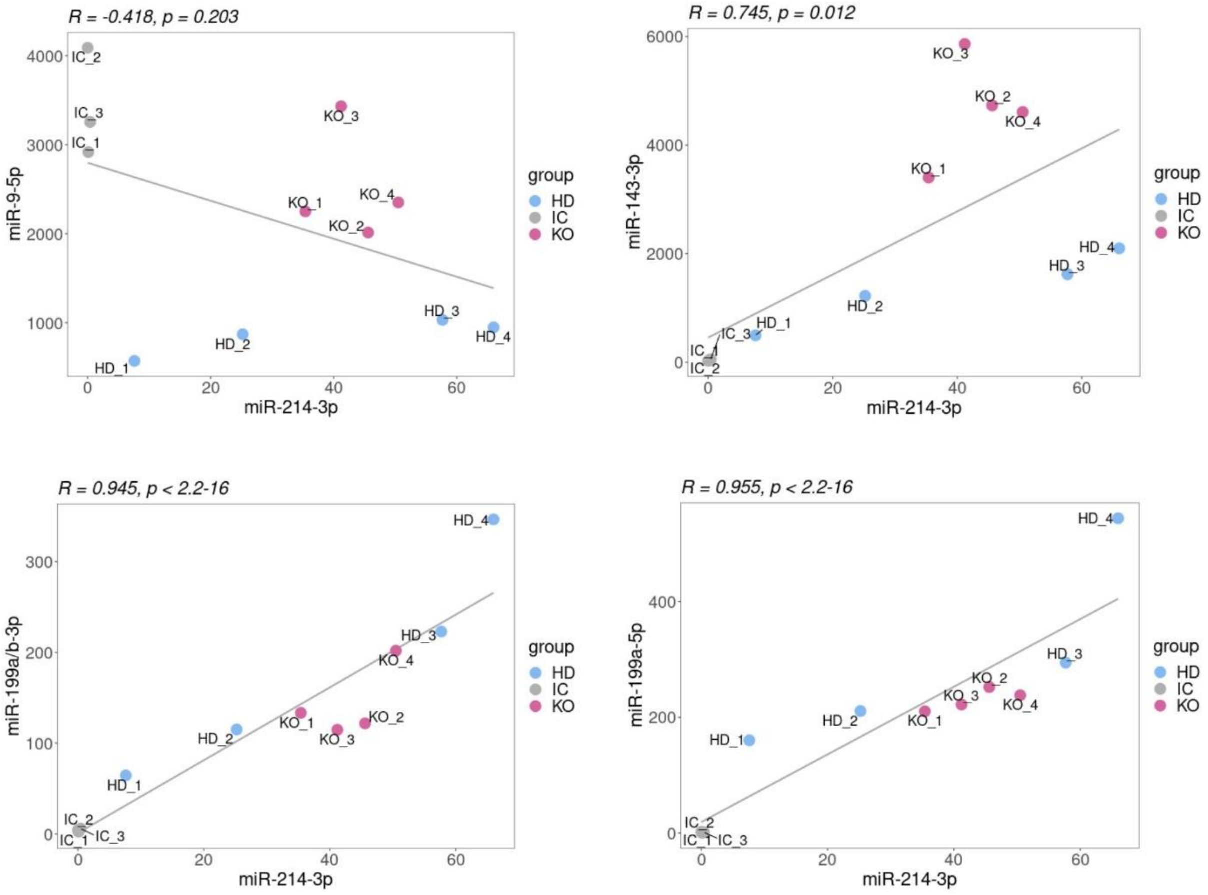

Supplementary Figure 4. Correlation of expression of selected TFs and miRNAs in IC1, HD and KO NSCs

Correlation plots for selected gene pairs were generated using ggplot2 v. 3.4.4 R package. Scatter plots present TPM expression values across all samples for selected gene pairs. Presented regression lines were added using the *geom_smooth* function (ggplot2 package) and Spearman correlation coefficients were calculated using the *cor.test* function (*stats* R package).

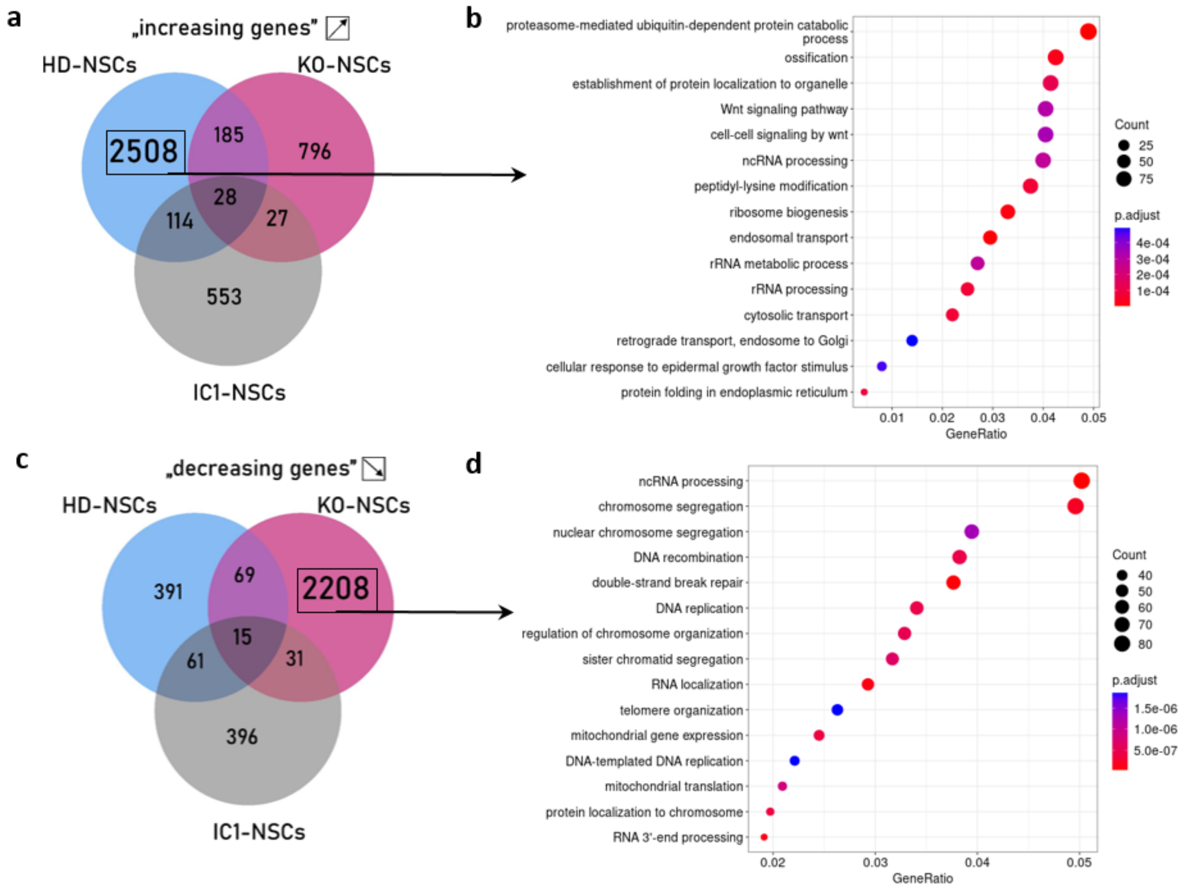

Supplementary Figure 5. Increasing and decreasing gene expression over time in NSC culture

(**a**) Venn diagram showing the number of genes with increasing expression in IC1, HD and KO cells based on RNA-seq data from 3 (for IC) or 4 (for HD and KO) time points (subsequent passages) of NSC culture. (**b**) GO biological process (GO- BP) enrichment analysis for genes classified as "increasing" only in HD-NSCs. (**c**) Venn diagram analogous as in (a) for genes with decreasing expression. (**d**) GO-BP enrichment analysis for genes classified as "decreasing" only in KO-NSCs.

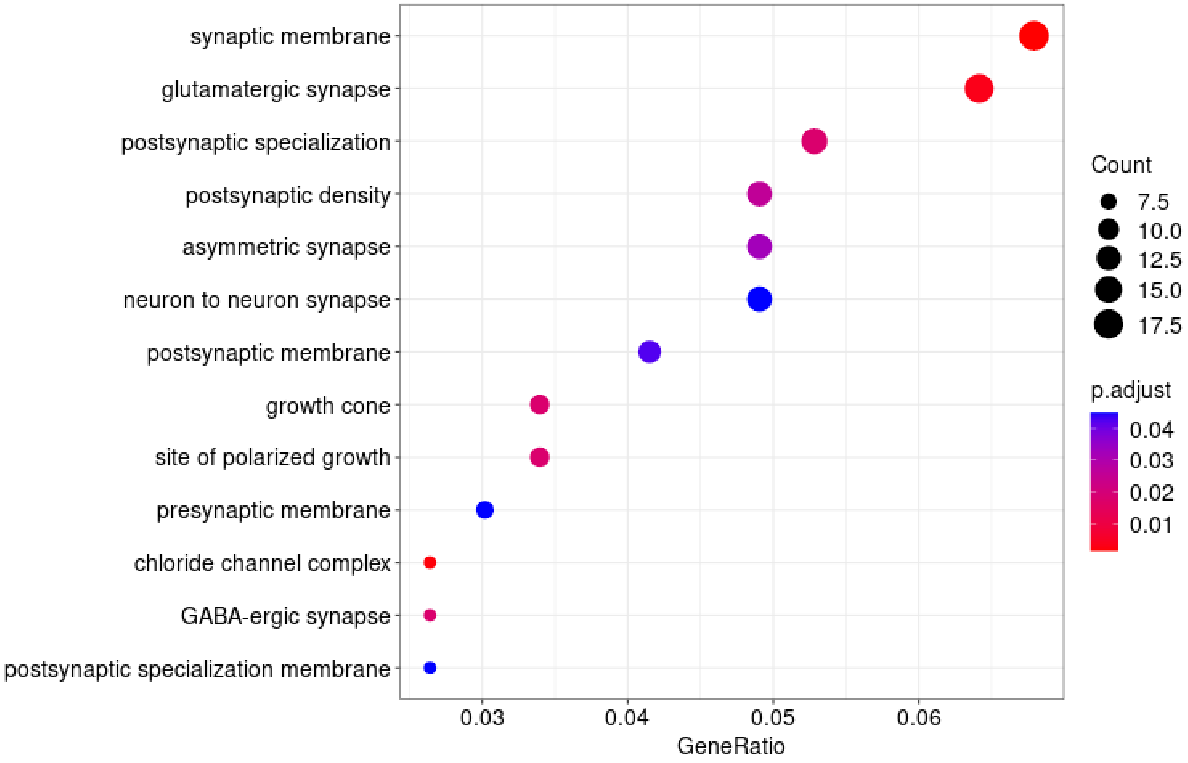

Supplementary Figure 6. GO enrichment analysis for genes classified as "increasing" in control NSCs

GO biological processes (BP) enrichment analysis was performed using a clusterProfiler R package. The significantly enriched GO-BP categories are listed and sorted by significance.

The y-axis is GO-BP term, and the x-axis shows the gene ratio representing the proportion of enriched genes in a GO term over the number of genes in the inputted gene list.

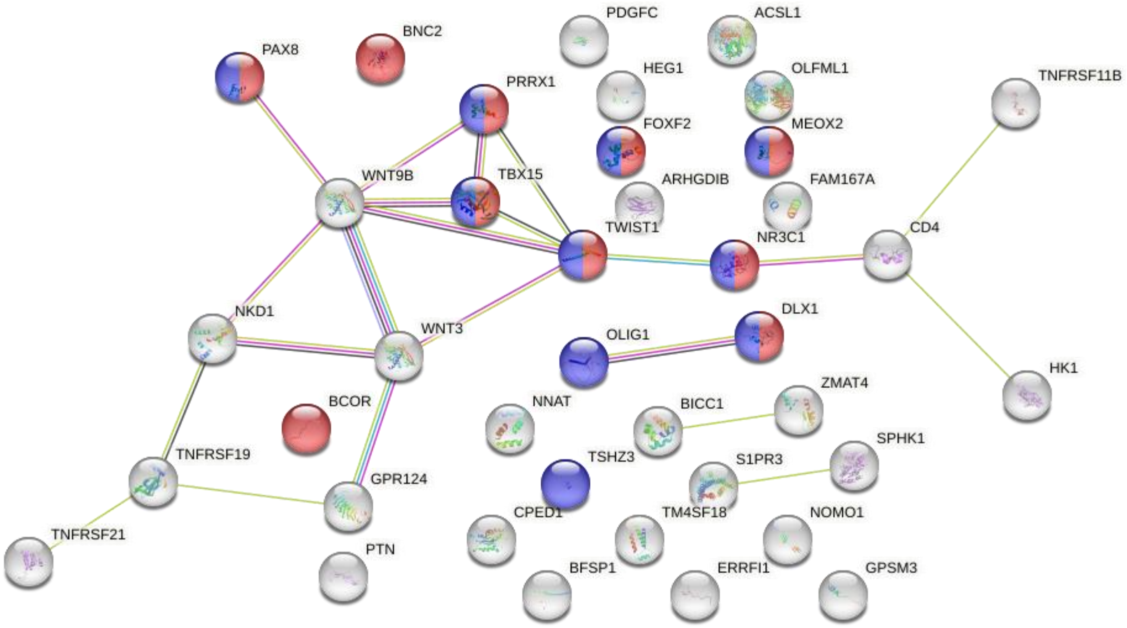

GO-term	description	FDR
GO:0000977	RNA polymerase II transcription regulatory region sequence-specific DNA binding	0,0215
GO:0000981	DNA-binding transcription factor activity, RNA polymerase II-specific	0,0265

Supplementary Figure 7. Enrichment of TFs that are associated with polymerase II in HD among „increasing genes" unique for HD-NSCs

Prediction of the PPI network was conducted using the STRING v11.5 database.

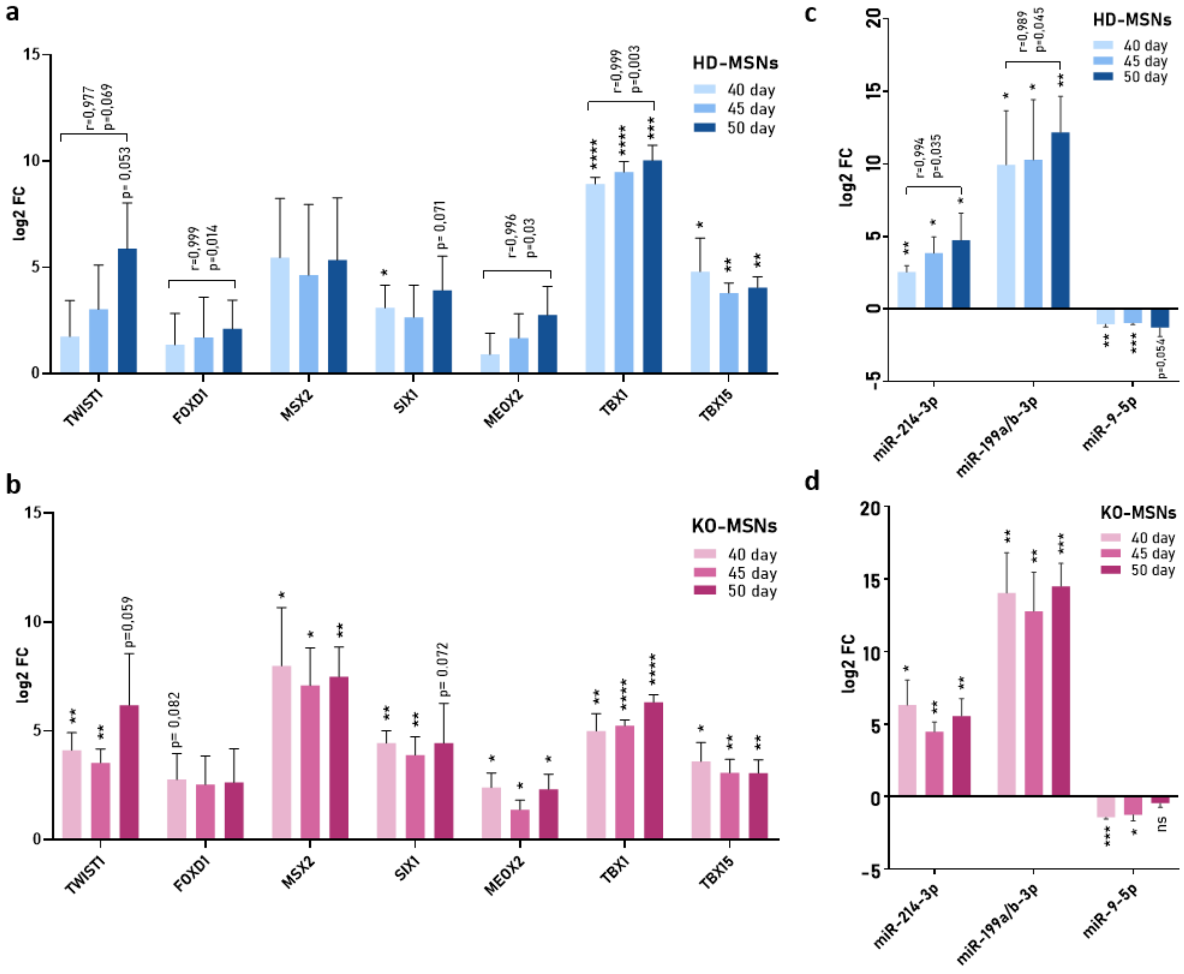

Supplementary Figure 8. Deregulation of selected TFs and miRNAs during the differentiation of HD-MSNs and KO-MSNs

(**a**, **b**) Relative expression of the selected TFs in 40-, 45- and 50-day-old HD-MSNs (a) and KO-MSNs (b) was analyzed using RT–qPCR. Reference genes: *EEF2* and *RPLP0*. (**c**, **d**) Relative expression of miR-214-3p, miR-199a/b-3p and miR-9-5p in 40-, 45- and 50-day-old HD-MSNs (c) and KO-MSNs (d) was assessed using RT–qPCR. Reference miRNAs: miR-16 and miR-92a. FC was calculated using the *delta-delta Ct* method and is shown as the log2 relative to that of IC2-MSNs at each of the time points; for example, 40-day HD-MSNs/KO-MSNs were referred to as 40-day IC2-MSNs. Statistical analysis was performed using multiple *t* tests, (a) and (b), followed by the Holm-Sidak post hoc test for (c) and (d). *0.01<p<0,05; **p<0.01; ***p<0.001; ****p <0.0001. r-Pearson correlation coefficient.
