# Supplementary material for: HTT loss-of-function contributes to RNA deregulation in developing Huntington’s disease neurons
Source: Cell Biosci. 2025 Jul 9;15:100. doi: 10.1186/s13578-025-01443-5 (PMC12239503; doi:10.1186/s13578-025-01443-5)
Supplement: Supplementary file 4 — Supplementary Material 4: Supplementary Table 2. A list of primers used for RT-qPCR. [file 13578_2025_1443_MOESM4_ESM.docx]

**Supplementary Table 2.** A list of primers used for RT-qPCR

| **Name** | **Forward (5'-3')** | **Reverse (5'-3')** |
| --- | --- | --- |
| *PAX6* | TGCTCCGGCATGAAATATACTA | GTCTCCAAATGTGCAGCAAC |
| *SOX1* | ACCAGGCCATGGATGAAG | CTTAATTGCTGGGGAATTGG |
| *SOX2* | CAAAAATGGCCATGCAGGTT | AGTTGGGATCGAACAAAAGCTATT |
| *RPLP0* | CATATCCGGGGGAATGTGGG | CAGCAGCTGGCACCTTATTG |
| *EEF2* | TCATCGAGGAGTCGGGAGAG | ACGACCGGGTCAGATTTCTTG |
| *TWIST1* | TACGCCTTCTCGGTCTGGAG | TTCTCTGGAAACAATGACATCTAGG |
| *FOXD1* | CGCTCGAGGAAGAAGGTAGG | GAGGAGCGAACAAAACACCG |
| *SIX1* | AGGTCAGCAACTGGTTTAAGAACC | GAGGAGAGAGTTGGTTCTGCTTG |
| *MSX2* | CGGAAAATTCAGAAGATGGAGCG | CGGCTTCCGATTGGTCTTGTGT |
| *MEOX2* | TCTCACCAGACTGAGGCGATAC | TCCACTTCATCCGCCTGTTTTGG |
| *TBX1* | TGGACCCACGCAAAGATAGC | TGAGCTGCGTGATCCGATG |
| *TBX15* | CTGGATGAGACAGGTGGTCAGT | GGTGAAAGGTCACTGCTGAAGTC |
