# Supplementary material for: HTT loss-of-function contributes to RNA deregulation in developing Huntington’s disease neurons
Source: Cell Biosci. 2025 Jul 9;15:100. doi: 10.1186/s13578-025-01443-5 (PMC12239503; doi:10.1186/s13578-025-01443-5)
Supplement: Supplementary file 13 — Supplementary Material 13: Supplementary Text and Methods [file 13578_2025_1443_MOESM13_ESM.docx]

**SUPPLEMENTARY TEXT**

To explore the phenomenon of progressive molecular changes in HD, we performed an additional bioinformatics analysis of our RNA-seq data, which revealed genes whose expression increased or decreased over time in HD, KO and control NSCs (lists of genes are provided in Table S7 and S8). For each cell line, differences in the TPM values were calculated between three (for IC1) or four (for HD and KO-NSC) time points, and specific criteria (described in Supplementary Methods) were applied to categorize genes as “increasing” or “decreasing” (Fig. S5).

The HD model exhibited the highest number of “increasing” genes (2835, representing 67% of ”increasing” genes in all NSC lines), including 2508 genes that were unique to HD-NSCs (which constituted 88.5% of “increasing” genes in this cell line) (Fig. S5a). The number of genes whose expression increased in the IC1-NSCs and KO-NSCs was considerably lower than that in the cells with mutation (722 and 1036, respectively). As expected, GO analysis revealed that in control cells (IC1-NSCs), “increasing” genes were associated mainly with neural differentiation (Fig. S6). The overlap between KO or HD and IC1 was relatively small (5.0% of HD and 5.3% of KO “increasing” genes), which might indicate disruptions of neural differentiation in cells with mutation and in those without *HTT* expression (Fig. S5a). To classify genes whose expression increased specifically in HD cells, we performed GO enrichment analysis on a set of 2508 genes specific to HD-NSCs. Among those genes, we found enrichment in 274 different biological processes (the top 15 are plotted in Fig. S5b; all the GO terms are presented in Table S9), i.e., processing different types of RNAs, posttranslational modifications of proteins, and Wnt pathway regulation. Moreover, PPI analysis (STRING) revealed enrichment of TFs that are associated with polymerase II, which confirmed our previous observation (Fig. S7).

Regarding “decreasing” genes, we identified the highest number of these genes in the KO-NSC model. Precisely, we found 2323 genes with decreasing expression in KO-NSCs (which constituted 73% of “decreasing” genes in all cell lines) (Fig. S5c). Most of these genes were unique to the KO model (2208 genes which accounted for 95% of “decreasing” genes in this cell line). In the IC1 and HD models, a substantially lower number of genes with decreasing expression was identified (503 and 536, respectively) (Fig. S5c). In the KO model, “decreasing” genes were classified as being associated with ncRNA processing, chromosome segregation and DNA recombination (Fig. S5d, all the GO terms are presented in Table S9). There was no substantial overlap between the two most numerous groups, “increasing” genes in HD-NSCs and “decreasing” genes in KO-NSCs (RF = 1.1, p <0,091), indicating dissimilarity in progressive changes in gene expression in these models.

Regarding selected TFs, our RNA-seq results revealed interesting differences between cells with *HTT* mutation and those without *HTT* expression, i.e., in HD-NSCs but not in KO-NSCs there was a tendency for the expression of several TFs (*TWIST1, MSX2, MEOX2,* and *TBX1*) to increase over time (with subsequent passages of NSCs) (Table S10a). This observation was confirmed by RT‒qPCR for these TFs (Table S10b). To further study the time-dependent increase of the selected TFs expression identified in HD-NSCs, we analyzed their levels in MSN-like cells at three time points of differentiation (Fig. 1b). In three independent differentiation experiments, We observed a trend toward increasing expression of *FOXD1, MEOX2* and *TBX1* (as well as a result that was close to statistically significance for *TWIST1*) in HD-MSNs but not in KO-MSNs (Fig. S8a, b). Next, we evaluated miRNA expression in the same neuronal samples and observed a trend toward increasing upregulation of miR-214 and miR-199 (high R for both miRNAs) in HD-MSNs (Fig. S8c). No such trend was observed in the KO-MSNs (Fig. S8d).

**SUPPLEMENTARY METHODS**

**Differentiation of iPSCs into NSCs**

iPSCs were grown in StemFlex (Gibco) medium on a Geltrex (Gibco)-coated 6-well plate until they reached 70–80% confluence. Then, the iPSCs were dissociated into single-cell suspensions by incubation with Accutase (STEMCELL Technologies) for 5-7 min (at 37°C). The cells were counted using a TC20 Automated Cell Counter (Bio-Rad), resuspended at a concentration of 1×10^6^ cells/mL, and seeded on Geltrex-coated plates in STEMdiff Neural Induction Medium supplemented with SMADi and 10 nM Y-27632 (a ROCK inhibitor) (all reagents from STEMCELL Technologies). For further cultivation, the cells were detached using Accutase, and after the third passage, they were grown in STEMdiff Neural Progenitor Medium (STEMCELL Technologies). After the fourth passage, the expression of the markers *SOX1, SOX2, PAX6*, and *NES* was confirmed by immunocytochemistry (ICC) (data not shown).

**Differentiation of NSCs into neuronal cells**

NSCs were grown in STEMdiff Neural Progenitor Medium (NPM) until they reached full confluence and then dissociated into single-cell suspensions by incubation with Accutase. The collected cells were passaged at a 1:5 ratio onto a Geltrex-coated 6-well plate in NPM supplemented with 10 nM Y-27632. The next day, the NPM was exchanged for lateral ganglionic eminence (LGE) pattering medium (N2B27 supplemented with 25 ng/mL recombinant human activin A) (STEMCELL Technologies). The medium was changed every day until day 10 when the LGE progenitors were passaged for terminal differentiation into MSNs. Then, the cells were passaged with Accutase at a ratio of 1:5 onto PDL/laminin-coated 6-well and 12-well plates (for ICC) in LGE pattering medium supplemented with 10 nM Y-27632. The next day, the LGE pattering medium was exchanged for terminal differentiation medium (N2B27 supplemented with 10 ng/mL recombinant human BDNF and 10 ng/mL recombinant human GDNF) (both from STEMCELL Technologies).

**RNA-seq libraries preparation and reads analysis**

Sequencing libraries were generated using the KAPA RNA HyperPrep Kit with RiboErase (HMR) (Kapa Biosciences) and IDT adapters for Illumina TruSeq DNA UD Indexes (96 Indexes, 96 Samples) for total RNA or using the TruSeq Small RNA Library Prep (Illumina) for miRNAs. The quality and quantity of the sequencing libraries were analyzed using a Bioanalyzer 2100 (Agilent) with a High Sensitivity DNA Kit (Agilent) and a Kapa Library Quantification Kit (Kapa Biosciences).

Quality reports were generated with FastQC v0.11.9. The reads were subjected to quality filtering and adapter trimming using BBDUK 2 v. 37.02. rRNAs mapped reads were removed following alignment with bowtie2 v. 2.3.5.1, with the -X 1000 parameter (for mapped read distance) and the *--un-conc* parameter. The expression values were obtained with RSEM v. 1.3.1, using default settings and bowtie2 as an alignment method. The ENSEMBL v. 102 and GRCh38 genome assembly were used as references. Differential expression analysis was performed using DESeq2 v. 1.30.0.

**Bioinformatics analysis of genes whose expression increased or decreased over time**

To select genes whose expression increased or decreased with time in NSCs according to RNA-seq data, we used the following criteria: (I) genes with TPM values below 1 at each time point were excluded; (II) genes that were classified as “increasing” or “decreasing” showed a consequent increase or decrease in TPM values, respectively, at subsequent passages; and (III) genes whose expression exhibited at least a 25% change in expression between the first and last time points were included. The percentage change in expression was calculated as the difference in the expression values at the first and last time points divided by the initial expression value and multiplied by 100%. The genes were sorted starting from the genes with the greatest changes in expression. GO enrichment analysis was conducted on selected sets of "increasing” and “decreasing” genes using the *enrichGO* function from the clusterProfiler v. 4.10.0 R package. Significantly enriched GO terms were identified through Benjamini–Hochberg correction (cutoff padj < 0.05, q value < 0.05). The results were sorted by GeneRatio and visualized using a *dotplot* (enrichplot v. 1.22.0 R package).
